# Evolutionary dynamics of *Euphorbia carniolica* suggest a complex Plio–Pleistocene history of understorey species of deciduous forest in southeastern Europe

**DOI:** 10.1111/mec.17102

**Published:** 2023-08-26

**Authors:** Philipp Kirschner, Eliška Záveská, Karl Hülber, Johannes Wessely, Wolfgang Willner, Peter Schönswetter, Božo Frajman

**Affiliations:** ^1^ Department of Botany University of Innsbruck Innsbruck Austria; ^2^ Faculty of Agricultural, Environmental and Food Sciences Free University of Bozen‐Bolzano Bolzano Italy; ^3^ Institute of Botany of the Czech Academy of Sciences Průhonice Czechia; ^4^ Department of Botany and Biodiversity Research University of Vienna Vienna Austria

**Keywords:** Alps, angiosperms, demographic modelling, forest understorey, glacial refugia, phylogeography

## Abstract

Deciduous forests form the dominant natural vegetation of Europe today, but were restricted to small refugia during Pleistocene cold stages, implying an evolutionary past shaped by recurrent range contractions and expansions. Cold‐stage forest refugia were probably widespread in southern and central Europe, with the northwestern Balkan Peninsula being of particular importance. However, the actual number and location of deciduous forest refugia, as well as the connections between them, remain disputed. Here, we address the evolutionary dynamics of the deciduous forest understorey species *Euphorbia carniolica* as a proxy for past forest dynamics. To do so, we obtained genomic and morphometric data from populations representing the species' entire range, investigated phylogenetic position and intraspecific genetic variation, tested explicit demographic scenarios and applied species distribution models. Our data support two disjoint groups linked to separate refugia on the northwestern and central Balkan Peninsula. We find that genetic differentiation between groups started in the early Pleistocene via vicariance, suggesting a larger distribution in the past. Both refugia acted as sources for founder events to the southeastern Alps and the Carpathians; the latter were likely colonised before the last cold stage. In line with traditional views on the pre‐Pleistocene origin of many southeastern European deciduous forest species, the origin of *E*. *carniolica* was dated to the late Pliocene. The fact that *E*. *carniolica* evolved at a time when a period of continuous forestation was ending in much of Eurasia provides an interesting biogeographical perspective on the past links between Eurasian deciduous forests and their biota.

## 1 INTRODUCTION

At present, deciduous forests are the dominant natural vegetation of temperate Europe (Ellenberg & Leuschner, 2010). Most of the current distribution of deciduous forests in Europe was unsuitable for tree growth during the Pleistocene cold stages such as the Last Glacial Period (LGP; 115–12 ka bp). This implies a dynamic history of deciduous tree species and associated forest understorey species, including large‐scale and rapid postglacial range expansions from refugial areas (Leroy, 2007). Based on palaeobotanical and genetic evidence, the northwestern Balkan Peninsula was of outstanding importance for the Holocene recolonisation of central and northern Europe and for southward expansion in the western Balkan Peninsula by European beech (*Fagus sylvatica* L.), the most abundant deciduous European tree species (Magri et al., 2006).

Refugia of deciduous forests dominated by beech were also corroborated by floristic data (Jiménez‐Alfaro et al., 2018; Willner et al., 2009), and some refugia outlined by Magri et al. (2006) were shown to harbour unique communities of narrowly distributed forest understorey species (from here on ‘deciduous forest understorey species’, DFUS). Especially the northwestern Balkan Peninsula is rich in DFUS such as *Anemone trifolia* L., *Cardamine trifolia* L., *Euphorbia carniolica* Jacq., *Hacquetia epipactis* (Scop.) DC., *Lamium orvala* L. and *Omphalodes verna* Moench. From there, the ranges of these DFUS continuously extend towards the adjacent Alps and the western Balkan Peninsula, but also include disjoint areas in Central Europe and/or the Carpathians. Traditionally, and based on this distribution, many of these species have been attributed to the ‘illyricoid floristic element’ (referring to the ancient Roman province of Illyria in the western Balkan Peninsula; ‘illyricoid species’ from here on; Horvat et al., 1974; Marinček, 1995; Trinajstić, 1992; Turrill, 1929). Trinajstić (1992) speculated that illyricoid species are ‘Tertiary’ palaeoendemics of pre‐Pleistocene origin that ‘survived the ice age in some refugia with mesophilous vegetation in between the southeastern Alps and the northwestern Dinaric Alps’, and colonised areas with temperate forests from there, resulting in their present disjunct distributions. However, neither the pre‐Pleistocene origin nor the spatiotemporal diversification of extant disjunctions of illyricoid DFUS has been tested or explored in detail, and, in contrast to temperate tree species such as beech, only little is known about their biogeographic history.

Available data suggest congruence of glacial refugia and postglacial migration routes between European beech and associated DFUS. For instance, for *Cyclamen purpurascens* Mill., the foothills of the Southern Limestone Alps, the northwesternmost Balkan Peninsula and possibly the Western Carpathians were potential LGP refugia (Slovák et al., 2012), which is largely concordant with refugia inferred for beech and other temperate tree species (Magri et al., 2006; Magyari, 2002). Such congruence was also suggested for the DFUS *Hordelymus europaeus* (L.) Jess., however, solely supported by plastid haplotype distribution (Dvořáková et al., 2010). Likewise, refugia in the Southern Limestone Alps and the adjacent Balkan Peninsula were supported for the DFUS *Hacquetia epipactis* (Scop.) DC (Urbaniak et al., 2018) and *Knautia drymeia* Heuff. (Rešetnik et al., 2016), and recently also for *Helleborus niger* L. (Záveská et al., 2021). In addition, it has been shown that different parts of the Carpathians provided suitable cold‐stage refugia for the long‐term survival of forest species (Bálint et al., 2011; Mráz & Ronikier, 2016; Ronikier, 2011). Evidently, most DFUS studied to date share a common cold‐stage refugium in the northwesternmost Balkan Peninsula, but possibly survived the LGP also in other, species‐specific refugia.

The spurge *E. carniolica* Jacq. is a DFUS with illyricoid distribution that spans disjunct areas at the southern margin of the Southern Limestone Alps, the northwestern and central Balkan Peninsula, as well as the Southern and Eastern Carpathians, including the Apuseni Mountains (Horvat et al., 1974; Trinajstić, 1992; Zimmermann et al., 1924). It is a typical forest understorey species that is primarily associated with beech (Török et al., 1989) with marginal habitats in the understorey of forests dominated by hop‐hornbeam (*Ostrya carpinifolia* Scop.) or Scots pine (*Pinus sylvestris* L.) as well as in subalpine grassland (Horvat et al., 1974; Pignatti et al., 2019; Zimmermann et al., 1924). *Euphorbia carniolica* is a diploid species with 16 chromosomes (Polatschek, 1971). Phylogenetically, it belongs to *E*. sect. *Helioscopia* Dumort and was resolved—together with its sister species *E. altaica* Ledeb.—as one of the sections' earliest diverging lineages (Riina et al., 2013). *Euphorbia altaica* is endemic to Siberia at more than 4000 km distance from the easternmost occurrences of *E*. *carniolica*, where it grows in subalpine grassland as well as in montane forests and grasslands (Prokhanov, 1949). The onset of diversification of *E*. sect. *Helioscopia* was dated to the early Miocene 19.8 Ma (highest posterior densities, HPD: 13.4–26.8 Ma; Horn et al., 2014), suggesting an old, pre‐Pleistocene origin of the *E. altaica*–*E. carniolica* lineage.

Here, by disentangling the spatiotemporal diversification of *E*. *carniolica*, we aim to contribute to the underexplored biogeographic history of deciduous forests in southeastern Europe. To this end, we applied multiple analyses based on genomic single nucleotide polymorphisms (SNPs) inferred from restriction‐site associated DNA sequencing (RADseq) and plastid DNA sequences to a range‐wide sampling of populations, and combined them with retrospective species distribution modelling. To set the spatial diversification in a temporal context, molecular dating analyses based on internal transcribed spacer (ITS) sequence data and a broad sampling of outgroup taxa were employed. After delimiting genomically coherent groups, we used explicit demographic model testing to reconstruct the history of the inferred genetic groups and the patterns of range expansion.

We tested three explicit hypotheses. (1) *E. carniolica* originated well before the Pleistocene as postulated—but rarely tested—for illyricoid species (Trinajstić, 1992; Turrill, 1929). (2) *E*. *carniolica* persisted in multiple glacial refugia, rather than a single refugium in the north‐western Balkan Peninsula (Trinajstić, 1992), which also facilitated formation of divergent lineages. We aim to explore the processes governing this lineage divergence that are either long‐distance dispersal, diffusion or vicariance. (3) Phylogenetic divisions in *E. carniolica* were accompanied by morphological and ecological differentiation; we tested this hypothesis using multivariate morphometrics and lineage‐specific niche modelling.

## 2 MATERIALS AND METHODS

### 2.1 Plant material

We covered 93 populations of *E. carniolica* that represent the species' entire range as indicated in the literature (e.g. Govaerts et al., 2000; Meusel et al., 1978; Nikolić, 2021; Poldini, 2002; Data S1, Figure S1). Specimens from these populations were used for morphometrics and genome size measurements, and locality data were used for niche modelling. From 69 populations, fresh leaves were sampled and dried in silica gel for DNA extraction, and voucher specimens were deposited at IB (Herbarium of the Department of Botany of the University of Innsbruck). A minimum distance of 5 m between sampled individuals was maintained to avoid sampling clones. *Euphorbia angulata* Jacq., which is one of the many species included in the most species‐rich clade of *E*. sect. *Helioscopia*, phylogenetically relatively distant to but co‐occurring with *E. carniolica* (Riina et al., 2013), was collected as an outgroup for phylogenomic analyses as no silica gel‐dried material of *E. altaica* was available (Data S1).

### 2.2 DNA extraction

Total genomic DNA was extracted from dried leaf tissue (ca. 10 mg) using a modified CTAB protocol (Tel‐zur et al., 1999) that included additional sorbitol‐washing steps (Frajman & Schönswetter, 2011), and purified with the NucleoSpin gDNA clean‐Up kit (Macherey‐Nagel). The DNA concentration was measured using a Qubit 4 fluorometer (ThermoFisher Scientific).

### 2.3 Sequencing of ITS and plastid DNA regions

ITS and the plastid *trnT*–*trnF* (TrnTF hereafter) region were sequenced to provide the general phylogenetic framework of *E*. sect. *Helioscopia* and infer the phylogenetic position of *E*. *carniolica*. This enabled divergence time estimation using published node ages from Euphorbia (Horn et al., 2014). In addition, the plastid *ndhF*–*trnL* region was sequenced to infer phylogeographic patterns within *E. carniolica*, complementary to genome‐wide RAD sequencing. Primers and amplification conditions for all sequenced regions are given in Table S1. ITS was amplified and sequenced for one individual from nine populations throughout the distribution range of *E. carniolica* (Data S1) as described by Frajman and Schönswetter (2011). Six additional species from *E*. sect. *Helioscopia* were sequenced, and 85 sequences from this section and its sister *E*. sect. *Holophyllum* (Prokh.) were taken from Genbank (Table S2), including one additional accession of *E. carniolica*. Final alignment length was 668 bp (sequences of *E. carniolica* were 641 bp long).

For one individual from 10 populations that represent the distribution range of *E. carniolica* (Data S1), *trnTF* was amplified and sequenced as in Frajman and Schönswetter (2011). In addition, five outgroup species were sequenced and 26 sequences from *E*. sect. *Helioscopia* (including an additional accession of *E. carniolica*) were taken from Genbank (Table S2). Sequences of *E. carniolica* were 1470–1492 bp, and the final alignment was 1750 bp long. After removing a poly‐A region between positions 683 and 686 and a poly‐T region between positions 1575 and 1585 from the alignment, we coded indels as binary characters by applying simple gap coding (Simmons & Ochoterena, 2000) in SeqState 1.25 (Müller, 2005).

Four plastid DNA regions totalling 7800 bp, that is *ndhF*–*trnL*, *rpoB*–*trnC*, *trnF*–*trnT* and *trnQ*–*trnK* (Shaw et al., 2007), were inspected for variability in *E. carniolica*. The *ndhF*–*trnL* spacer was most variable and was thus amplified and sequenced for one individual per population from 69 populations of *E. carniolica* (Data S1) for phylogeographic analyses, as described in Pahlevani and Frajman (2023). The sequences were 549 or 550 bp long.

Sequencing was done by Eurofins Genomics (Ebersberg). Contig assembly, sequence alignment and editing were done using Geneious Pro 5.5.9 (Kearse et al., 2012). GenBank accession numbers for *E. carniolica* are in Data S1.

### 2.4 Phylogenetic analyses of plastid *trn* *T* *F* and ITS sequences of *E.* sect. *Helioscopia*, including molecular dating

Maximum parsimony (MP) and MP bootstrap (MPB) analyses of ITS and *trnTF* were separately done using paup 4.0b10 (Swofford, 2002). In both cases, the most parsimonious trees were inferred heuristically with 100 replicates of random sequence addition, TBR swapping and MulTrees on. Swapping was performed on a maximum of 1000 trees (nchuck = 1000). All characters were equally weighted and unordered. Bootstrapping was done using full heuristics, 1000 replicates, TBR branch swapping, MulTrees option off and random addition sequence with five replicates. Bayesian analyses were performed using MrBayes 3.2.1 (Ronquist et al., 2012) applying the GTRI + Γ (ITS) and GTR + Γ (*trnTF*) substitution model proposed by the Akaike information criterion (AIC) inferred via MrAIC.pl 1.4 (Nylander, 2004). The *trnTF* alignment was partitioned into nucleotide and indel sets, and indels were treated as morphological data (Lewis, 2001). Settings for the Metropolis‐coupled Markov chain Monte Carlo process included four runs with four chains each (three heated using the default heating scheme) and ran simultaneously for 10,000,000 generations each. Trees were sampled every 1000th generation using default priors. Posterior probabilities (PP) of the phylogeny were determined from all trees whereas the first 1001 trees of each run were discarded as burn‐in. The performance of the analysis was checked in tracer 1.6.0 (Rambaut et al., 2018).

Based on the topology of the ITS phylogeny, we included two accessions of *E. altaica* and five of *E. carniolica* from all major clades, as well as all outgroup taxa in dating analyses. Divergence times were estimated using the pruned ITS alignment with beast 1.8.2 (Drummond et al., 2012), using a birth–death speciation prior (Gernhard, 2008) and GTRI + Γ substitution model and estimated base frequencies. A lognormal relaxed clock with a weakly informative prior on the clock rate (exponential with mean 0.001) was applied. Secondary calibration of the root was based on Horn et al. (2014). The root age prior was set to 23.4 Ma with a normally distributed standard deviation of 3.5 Ma. This corresponds to the median age and 95% HPD interval of the split between *E*. sect. *Holophyllum* (Prokh.) Prokh. and *E*. sect. *Helioscopia* (23.4 Ma, HPDs 16.1, 31.3; Horn et al., 2014). Two independent MCMC chains were run for 10,000,000 generations, saving trees and parameters every 1000 generations. The performance of the analysis was checked in tracer 1.6.0 (Rambaut et al., 2018); both the effective sample sizes (ESS > 200) and mixing were appropriate. Log and tree files from both runs were combined using Log Combiner after discarding 10% of each run as burn‐in, and a maximum clade credibility tree (MCCT) was then produced and annotated with Tree Annotator (both 1.8.2; Drummond et al., 2012). Trees were visualised with figtree 1.4.2 (Rambaut, 2014).

### 2.5 Phylogeographic analysis of plastid *ndhF* – *trnL* sequences of *E. carniolica*

A statistical parsimony network based on the *ndhF**–**trnL* alignment of *E. carniolica* was constructed in TCS (Clement et al., 2000), using a connection limit of 95 and treating a single gap as fifth character.

### 2.6 Library preparation, identification of RADseq loci and SNP calling

Single‐digest RADseq libraries were prepared using the restriction enzyme Pst1 (New England Biolabs) and following the protocol in Paun et al. (2016). In brief, 110 ng input DNA was enzymatically digested before ligation of 100 mM P1 adapters. Restricted fragments were sheared by sonication using a M220 Focused‐ultrasonicator (Covaris) to obtain a modal fragment size of 400 bp (range of 200–800 bp, peak in power: 50, duty factor 10%, 200 cycles per burst and treatment time 90 s at 20°C). Subsequently, individual P2 adapters were ligated followed by size selection and purification steps using SPRI beads (Beckman Coulter). Libraries were sequenced at the Vienna BioCenter Core Facilities on an Illumina Hiseq 2500 v4 and Illumina NovaSeq SP platform resulting in 100 bp single‐end reads.

Raw Illumina reads were demultiplexed, and quality filtered (only Phred >10 were retained) using BamIndexDecoder (available at https://github.com/wtsi‐npg/illumina2bam). SNPs were called using *denovo_map.pl* implemented in stacks 2.4 (Catchen et al., 2013). Initially, this pipeline was run on subsets of the data to assess parameters for an optimal loci yield (Paris et al., 2017). The settings ‐M 2 ‐m 5 ‐n 2 (−M: maximum bp difference between two stacks within a sample, −m: minimum coverage for a stack, −n: maximum bp difference between stacks to be considered as orthologous across samples) were suggested as optimal for SNP calling in *denovo_map.pl* (Catchen et al., 2013).

### 2.7 Bayesian clustering and phylogenetic analyses

For Bayesian clustering, SNPs were exported from the catalogue via *populations* in structure format using the *‐‐write‐single‐snp* flag to export a single SNP per fragment (to avoid including linked SNPs), and the *‐‐max‐obs‐het* 0.65 and *‐R* 0.8 flags to filter for paralogs and to exclude fragments missing in >80% of individuals respectively (Catchen et al., 2013). Clustering was done in structure 2.3.4 (Pritchard et al., 2000) using the admixture model including 69 populations with three populations each (except only two individuals in case of populations Ec17 and Ec18), for *K* (number of groups) ranging from 1 to 10, 10 replicates per *K* and 500,000 MCMC iterations after an initial burn‐in of 50,000 iterations. Hierarchical subclustering of the two main clusters using the same parameters assuming *K* 1–5 groups was done to further explore substructure within the data as suggested in Janes et al. (2017). To obtain information on the optimal number of *K* present within the data, log probability of data PrX|*K* and Δ*K* was calculated (Evanno et al., 2005; Pritchard et al., 2000).

Genetic variation was additionally visualised using a principal component analysis (PCA) based on the input file used for Bayesian clustering that included 205 individuals from 69 populations. The function *scaleGen* was used to calculate average frequencies at sites missing in the SNP matrix, before the function *dudi.pca* was used to obtain principal components (both included in the r package adegenet, Jombart & Ahmed, 2011). Populations were ordinated based on the first two principal components, and additionally colour‐coded according to the third principal component, using the function *colorplot* from adegenet (Jombart & Ahmed, 2011).

Phylogenetic relationships within *E*. *carniolica* were inferred in raxml 8.2.11 (Stamatakis, 2014), using *E. angulata* for rooting. Alignments were exported via *populations* using the *‐‐phylip_var_all* option, and the *‐‐max‐obs‐het* 0.65 and *‐R* 0.7 flags to filter for paralogs and to exclude fragments missing in >70% of individuals respectively (Catchen et al., 2013). Invariant sites were removed from the alignment using *ascbias.py* (https://github.com/btmartin721/raxml_ascbias). Twenty initial trees were computed under a Jukes–Cantor substitution model without rate heterogeneity and the Felsenstein correction for ascertainment bias (−m ASC_GRTCAT ‐‐JC69 ‐V ‐asc‐corr = felsenstein). The best scoring tree was bootstrapped using the frequency‐based stopping criterion (Pattengale et al., 2010).

### 2.8 Demographic modelling

To test divergence scenarios in *E. carniolica*, we used the software dadi that is based on diffusion approximation and utilises information from two‐dimensional joint site frequency spectra (2D‐JSFS, Gutenkunst et al., 2009). We used an established 2D‐JSFS analysis pipeline (Portik et al., 2017) and adapted python scripts (https://github.com/dportik/dadi_pipeline) to define 2D‐JSFS models, perform model fitting and execute plotting functions. To prepare 2D‐JSFS, vcf files were exported via *populations* using the ‐p 2 flag to only include the SNPs present in the targeted population pair, and the ‐‐single‐snp flag to avoid selection of linked SNPs (Catchen et al., 2013). *vcftools* (Danecek et al., 2011) was used to exclude SNPs that were missing in >25% of all individuals (*‐‐max‐missing* 0.75) and had a minimum mean coverage >10 (*‐‐min‐meanDP* 10). 2D‐JSFS were inferred and down projected using easySFS (https://github.com/isaacovercast/easySFS). Model parameter optimisation in the dadi_pipeline was done in four rounds with an increasing number of replications for each model (60, 70, 70, 80). Model evaluation was done using replicates with the highest likelihood to calculate AIC scores, ∆AIC scores, and Akaike weights (*ω*_*i*_; Burnham & Anderson, 2002); these measures were subsequently used to select the best‐fitting model.

For demographic modelling, the following three groups’ pairwise tests were done based on four a priori defined groups: *nBalk* versus *cBalk*, *Alp* versus *nBalk* and *cBalk* versus *Carp*. All four groups were resolved and supported by RADseq‐based phylogenetic analyses, Bayesian clustering and PCA (except for *Alp* that was only supported in the phylogeny; see Section 3). All Carpathian populations were summarised as subgroup *Carp*, and substructure was not further considered. To explore the evolutionary relationships between the constituents of these pairs, a predefined set of divergence models that allows differentiating between long‐term vicariance and founder events by incorporating directionality and initial size of the founding fraction was used (Charles et al., 2018; Záveská et al., 2021; details in Table S3). Directionality between the phylogenetically ancestral groups *nBalk* and *cBalk* was tested by comparing likelihoods of two independent modelling runs in which either population was defined as ancestral. For the comparisons *nBalk* versus *Alp* and *cBalk* versus *Carp*, *nBalk* and *cBalk* were defined as ancestral, and Alp and Carp as derived.

### 2.9 Relative genome size estimation

Relative genome size (RGS) was measured using flow cytometry as described by Stojilkovič et al. (2022). Briefly, nuclei of silica gel‐dried material of one to five individuals from all 82 populations of *E. carniolica* (43 from the *Northern Balkan–Alpine Group* and 39 from the *Central Balkan–Carpathian Group* (Data S1) as well as of fresh leaves of a reference standard were stained using 4′,6‐diamidino‐2‐phenylindole (DAPI). A CyFlow space flow cytometer (Sysmex Partec) was used to record the relative fluorescence of 3000 nuclei and FloMax software (Partec) was used to evaluate the results. RGS was calculated as the ratio between the values of the mean relative fluorescence of the sample and the standard using R 3.6.3 (R Core Team, 2022). Population‐wise RGS means and standard deviations were calculated (Data S1), and graphically summarised using the R package ggplot2 (Wickham, 2016). A Kruskal–Wallis H test (*kruskal.test*; R Core Team, 2022) was used to test for significant differences of RGS between the two genetic groups. This test was used because a Shapiro–Wilks test (*shapiro.test*; R Core Team, 2022) showed that RGS data were not normally distributed within the two groups.

### 2.10 Morphological differentiation

To assess differentiation between the two main genetic groups, morphometric analyses were done for one individual per population (Data S1). In total, 42 individuals from the *Northern Balkan–Alpine Group* and 35 individuals from the *Central Balkan–Carpathian Group* were analysed.

After initial inspection of the herbarium material, a list of 71 morphological characters was compiled, of which 52 characters were measured and 19 were calculated as ratios (Table S4). Stem characters were measured manually, whereas leaf characters were measured on scanned herbarium images using ImageJ (Abràmoff et al., 2004). Other characters (cyathium, fruit and seed as well as leaf trichome characters) were measured on images taken with a stereomicroscope Olympus SZX9 using the Olympus image analysis software analySIS pro. Cyathium and fruit characters were developed in 22 individuals of the *Northern Balkan–Alpine Group* and 12 individuals of the *Central Balkan–Carpathian Group*, and seeds were present in nine and two individuals respectively. When available, seed characters were scored on three seeds per voucher. Missing values were substituted with the character mean inferred for the corresponding genetic group.

Statistical analyses were done in SPSS 24.0 (IBM, SPSS Inc.). Correlation among metric characters was tested using Pearson and Spearman correlation. One character from each pair with a correlation coefficient >0.9 was excluded from further analyses, that is, the characters 2, 17, 22, 30, 33, 34, 41 and 42 (Table S4). After standardisation to zero mean and one unit variance, a PCA was done to visualise the data. Subsequently, discriminant analysis (DA) was performed to explore the morphological differentiation and to find characters best separating the genetic groups. PCA and DA were performed separately for vegetative characters as well as for cyathium and fruit characters. All morphometric data were used to describe both genetic groups and to produce an identification key. Due to the small number of seeds in the *Central Balkan‐Carpathian Group*, seed character data were only used in the descriptions of the two groups presented in Appendix 1.

### 2.11 Modelling lineage occurrences and analyses of climatic niches

Species distribution models (SDMs) were used to identify suitable areas of the two main allopatric lineages of *E. carniolica* (equivalent to the *Northern Balkan–Alpine Group* and the *Central Balkan–Carpathian Group*) under current and past climatic conditions. These models relate species' occurrences to environmental variables (the selection of variables is described in the Supplementary Material section ‘Bioclimatic variables and modelling lineage occurrences’). Occurrences of all sampled populations, and additional records from herbarium specimens (Data S1), were used as presence data. Both SDMs were parameterised within the BIOMOD framework (Thuiller et al., 2009; details in Supplementary Material section ‘Parametrisation of SDMs’).

Niche overlap between the *Northern Balkan–Alpine Group* and the *Central Balkan–Carpathian Group* was computed (Broennimann et al., 2012) and niche equivalency and similarity tests were employed to check if lineage‐specific niches were identical in their realised distributions in environmental space (Warren et al., 2008; details in Supplementary Material). Finally, niche breadth, that is, the area of an ellipse in environmental space, was calculated using the script ‘user_script_Nsp_1A.R‘(available at http://www.unil.ch/ecospat/home/menuguid/ecospat‐resources/tools.html). The diameters of this ellipse are defined by the variance of values (of a random set of cells) along each of the two PCA axes. All analyses were done in r 3.6.3 (R Core Team, 2022).

## 3 RESULTS

### 3.1 ITS and plastid *trnTF* phylogenies and divergence time estimation

**FIGURE 1:**
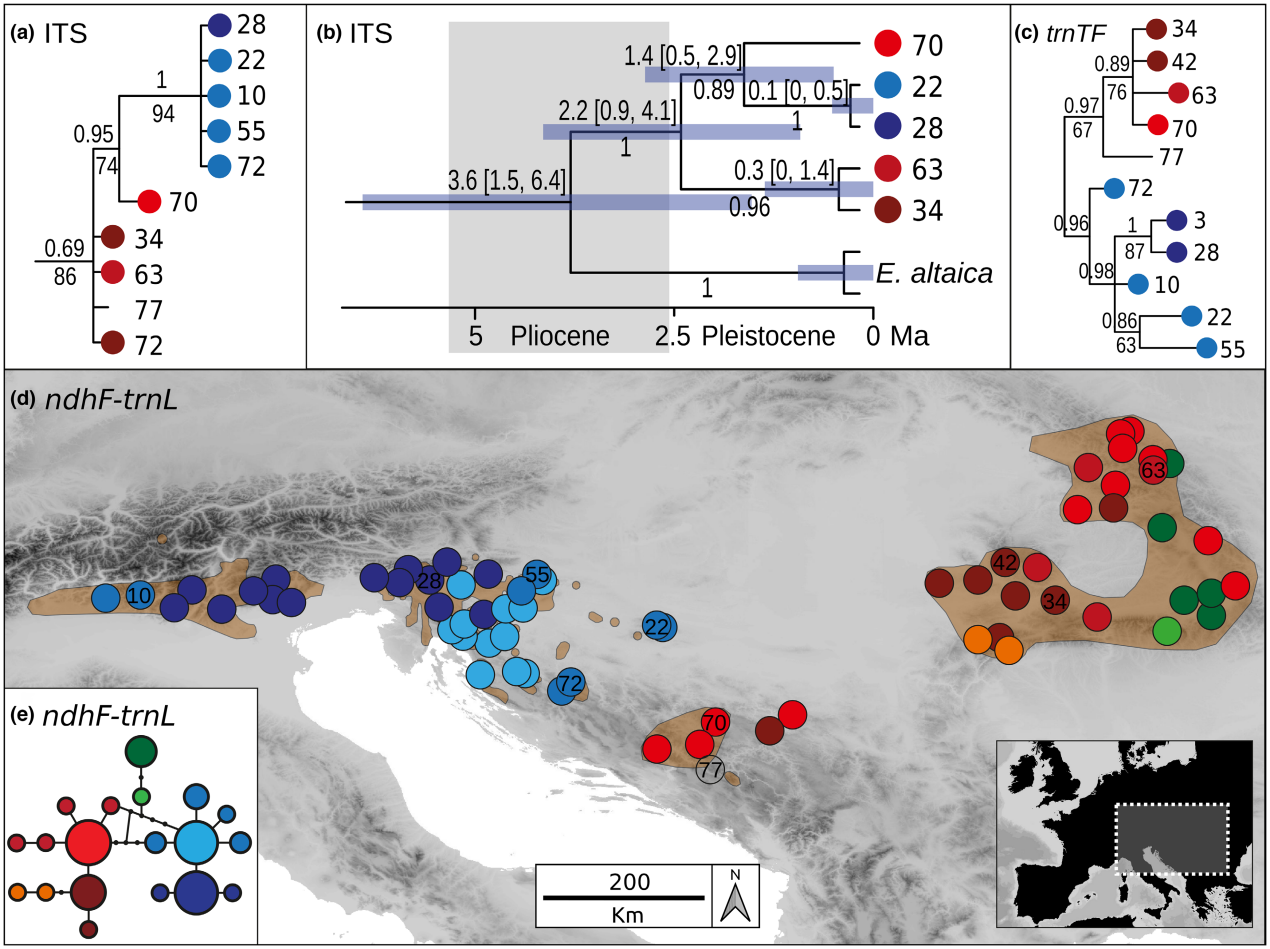
Internal transcribed spacer (ITS) and plastid DNA (*ndhF–trnL* and *trnT–trnF*) variation in *Euphorbia carniolica*. Population numbers correspond to Data S1. (a) Bayesian consensus phylogram inferred from ITS sequences of *E. carniolica*. Numbers above branches are posterior probabilities (PP), those below branches maximum parsimony bootstrap values (BS). The complete tree is in Figure S4. (b) Bayesian consensus chronogram (maximum clade credibility tree). Numbers below branches are PP, those above branches the median crown group age in millions of years, and the bars and the numbers in brackets correspond to 95% highest posterior densities (HPD) of the age estimates. The complete tree is in Figure S6. (c) Bayesian consensus phylogram inferred from plastid *trnT–trnF* sequences of *E. carniolica*. Numbers above branches are PP, those below branches BS values. The complete tree is in Figure S5. (d) Distribution of *ndhF–trnL* haplotypes retrieved by the analysis shown in E. Haplotypes not sampled are shown as small black dots. Light brown polygons indicate the species' distribution range according to Meusel et al. (1978) and supplemented with additional, more recent distribution data (Nikolić, 2021; Poldini, 2002). The insert shows the position of the study area in Europe. (e) Statistical parsimony network of *ndhF–trnL* haplotypes. The size of a circle is relative to the square root of a haplotype's frequency.

**FIGURE 2:**
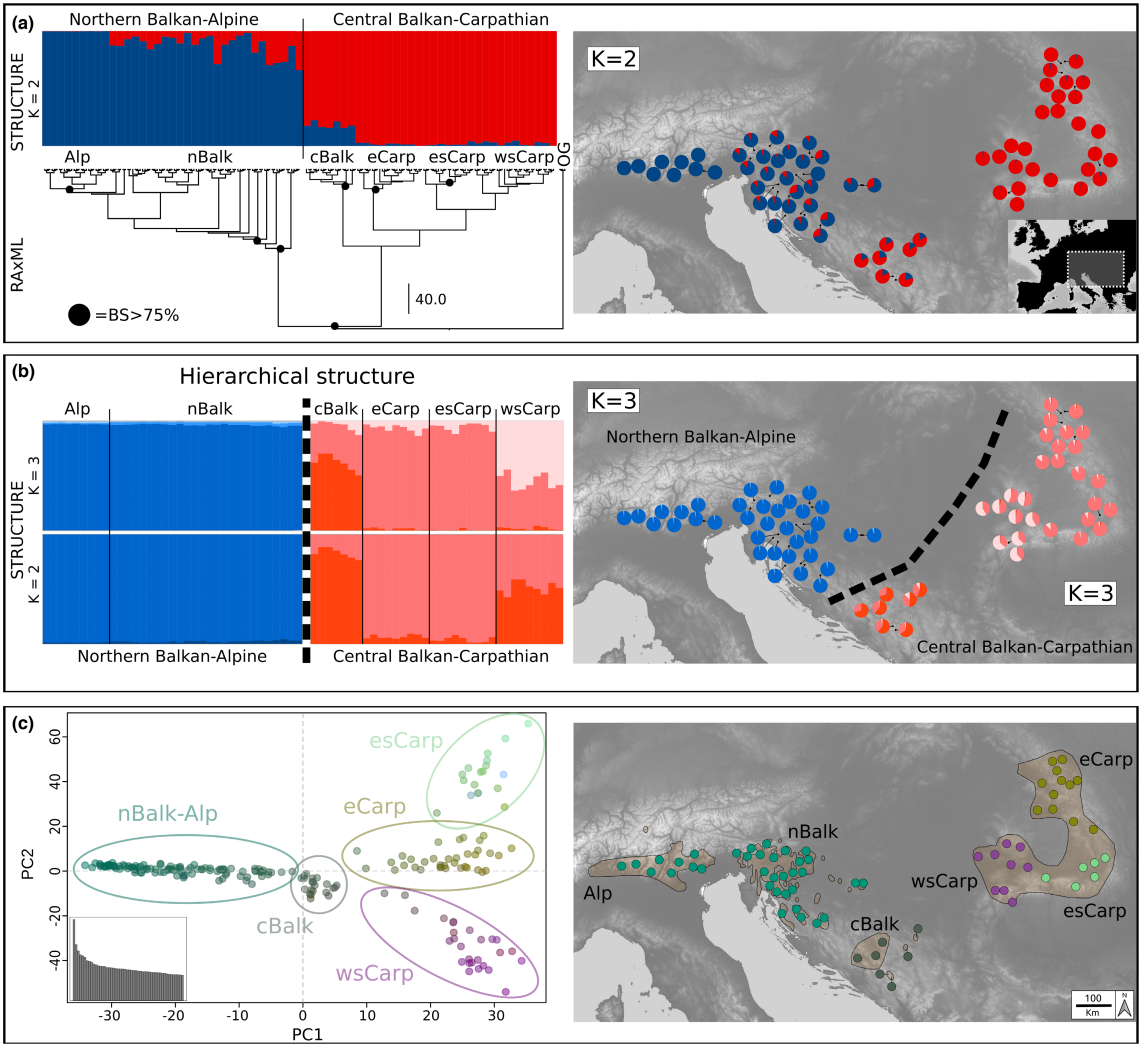
Phylogenetic relationships and genetic variation within *Euphorbia carniolica* based on restriction site‐associated DNA sequencing. (a) Phylogenetic tree inferred via maximum likelihood. Black circles above nodes highlight bootstrap support (BS) >75%. Barplots above the tree show the proportion of shared genetic variation inferred from Bayesian clustering for *K* = 2 groups; the same proportions are shown as pie charts in the map at the populations' sampling localities; overlapping pie charts were slightly shifted to aid legibility. (b) Proportion of shared genetic variation within each of the two main clusters shown in (a) inferred via groupwise Bayesian clustering for *K* = 2 and *K* = 3 groups. Proportions for *K* = 3 groups of both clusters are shown as pie charts in the map at the sampling localities. (c) Ordination of genomic variation along principal components (PC1: *x*‐axis, PC2: *y*‐axis, PC3: colour‐gradient); the barplot shows eigenvalues of the first 50 principal components. Ellipses in the ordination indicate the groups as in (a) and (b). Populations in the map are colour‐coded based on their group affiliation in the principal component analysis. Group names: *Alp*, Alpine group; *nBalk*, Northern Balkan group; *cBalk*, Central Balkan group; *eCarp*, Easter Carpathian group; *esCarp*, Eastern Southern Carpathian group; *wsCarp*, Western Southern Carpathian group. Light brown polygons indicate the distribution range of *E*. *carniolica* according to Meusel et al. (1978) and supplemented with additional, more recent distribution data (Nikolić, 2021; Poldini, 2002).

Bayesian and parsimony‐based phylogenetic trees based on ITS and *trnTF* were largely congruent for each marker and corresponded well in topology with previously published results (Figures S4 and S5; ITS: Riina et al., 2013, *trnTF*: Frajman & Schönswetter, 2011). In the ITS tree (Figure 1a), *E. carniolica* was resolved as monophyletic by parsimony (bootstrap, BS 86%; PP, 0.69) and sister (BS 100%; PP 1) to *E. altaica*. Within *E. carniolica*, four accessions in basal polytomy corresponded to the *Central Balkan–Carpathian Group* resolved by RADseq data and a clade (BS 94%, PP 1) containing five accessions corresponding to the *Northern Balkan–Alpine Group* (Figures 1a and 2). Compared to the RADseq phylogeny, the only incongruence was the placement of ITS accession BH70 in a group (BS 74%; PP 0.95) equivalent to the *Northern Balkan–Alpine Group* (BS 74%; PP 0.95), instead of the *Central Balkan–Carpathian Group* (Figures 1a and 2).

In the *trnTF* tree (Figure 1c; Figure S5), *E. carniolica* was split into two groups corresponding with the *Central Balkan–Carpathian Group* (BS 67%; PP 0.97) and the *Northern Balkan–Alpine Group* (PP 0.96) revealed by RADseq data (Figures 1c and 2). These two groups were resolved within a polytomy including several other species. *Euphorbia altaica* was clearly divergent from the clade containing *E. carniolica*.

The ITS chronogram (Figure 1b; Figure S6) was largely congruent with the ITS‐inferred topology (Figure S4). Only the early‐diverging lineages within *E*. sect. *Helioscopia*, including *E. carniolica*, that were in a polytomy in the ITS tree, appeared gradually diverging in the ITS chronogram, but these relationships received low support (PP <0.6). Inferred ages corresponded well with those in Horn et al. (2014) as the HPD intervals of corresponding groups were largely overlapping. *Euphorbia* sect. *Helioscopia* originated in the late Oligocene 24.0 Ma (HPD 17.1, 30.1) and started to diversify in the early Miocene 22.1 Ma (HPD 15.4, 28.6), when *E. coniosperma* Boiss. & Buhse originated. All other main clades, including the monophyletic lineage comprising *E. altaica* and *E. carniolica*, originated in the mid‐Miocene. The common ancestor of *E. altaica* and *E. carniolica* thus originated 14.1 Ma (HPD 8.9, 19.5), while the two species diverged in the mid‐Pliocene 3.6 Ma (HPD 1.5, 6.4). The onset of diversification within *E. carniolica* was dated to the early Pleistocene 2.2 Ma (HPD 0.9, 4.1), with the split between Central Balkan and Carpathian populations—that were in the chronogram monophyletic and not in a basal polytomy—and Northern Balkan–Alpine populations. Further diversification within the main lineages of *E. carniolica* continued throughout the Pleistocene.

### 3.2 Phylogeography of *E. carniolica* based on plastid sequences

Two main haplogroups within *E*. *carniolica* were also resolved by a haplotype network based on *ndhF**–**trnL* sequences that fully corresponded to the *Central Balkan–Carpathian Group* and the *Northern Balkan–Alpine Group* (Figures 1d,e and 2). Within the former group, 11 haplotypes were resolved and the haplotype diversity was largest in the Carpathians, where all 11 haplotypes were found. No regional differences were apparent in the latter group, in which eight haplotypes were resolved.

### 3.3 Intraspecific structure and phylogeography of *E. carniolica* based on RADseq data

A total of 210 samples was sequenced via RADseq (207 of *E*. *carniolica*, three of *E*. *angulata*). Prior to SNP calling, the average number of reads per sample was 0.79 million (SD ± 0.31) and the final SNP catalogue contained 316,418 loci composed of 28,260,735 sites with a mean coverage of 14.4 (SD ± 3.6). RADseq reads are available in the NCBI Short Read Archive BioProject PRJNA983383 (accession nos SAMN35724938–SAMN35725141, Data S1). Two individuals from populations Ec17 and Ec18 were excluded from subsequent analyses as they were identical with another sequence from the respective populations and possibly of clonal origin.

Bayesian clustering analyses were based on 12,824 unlinked SNPs. An optimal separation into two groups, termed *Northern Balkan–Alpine Group* and *Central Balkan–Carpathian Group*, was observed (Figure 2a) and remained stable when *K* = 2–10 groups were assumed (optimal *K* based on PrX|*K*: *K* = 2, and based on Δ*K*: *K* = 5; clustering results for *K* = 2–5 in Figure S2). Large‐scale admixture was detected in the transition area between both groups in the Balkan Peninsula, especially in the eastern and southernmost populations of the *Northern Balkan–Alpine Group* (Figure 2a), whereas both the easternmost Carpathian populations and the westernmost Alpine populations showed little or no admixture at all. Subclustering analyses of the two main groups revealed no subgroups in the *Northern Balkan–Alpine Group* (optimal *K* based on PrX|*K*: *K* = 2, and based on Δ*K*: *K* = 2). The *Central Balkan–Carpathian Group* contained distinct substructure (optimal *K* based on PrX|*K*: *K* = 3, and based on Δ*K*: *K* = 4) that was resolved by *K* = 2–5 while admixture proportions changed (Figure S2). Structure was best resolved and was biologically most plausible assuming *K* = 2 and *K* = 3 (Figure 2b). In both instances, the central Balkan populations (*cBalk*) and populations from the western Southern Carpathians (including the Apuseni Mts; *wsCarp*) formed distinct and admixed clusters (*K* = 2 and 3), and populations from the eastern Southern Carpathians (*esCarp*) and the Eastern Carpathians (*eCarp*) formed another cluster that did not show large‐scale admixture (Figure 2b).

A RAxML analysis based on 19,842 variant sites (Figure 2a; Figure S3) resolved *E*. *carniolica* as monophyletic (BS 100%) compared to the outgroup *E*. *angulata*. Within *E*. *carniolica* two lineages, corresponding with the *Northern Balkan–Alpine Group* and the *Central Balkan–Carpathian Group* of the Bayesian clustering analyses, were found (BS 100% and BS 54% respectively). The *Northern Balkan–Alpine Group* included a single supported lineage (BS 92%) composed of populations from the Southern Limestone Alps in Italy (*Alp*), and the remaining populations form the Northern Balkan Group (*nBalk*). The lineage equivalent to the *Central Balkan‐Carpathian Group* comprised several geographically coherent lineages that correspond with Bayesian clustering results, but only subgroups *cBalk* and *eCarp* were well supported (BS >75%; Figure 2a,b). The same structure was reflected by a PCA (Figure 2c; first two axes explaining 2.0% and 1.3% of total variation) that clearly resolved *cBalk* and the three Carpathian subgroups *eCarp*, *esCarp* and *wsCarp*, whereas the *Northern Balkan*–*Alpine Group* was coherent.

### 3.4 Demographic history within *E. carniolica* based on RADseq data

**FIGURE 3:**
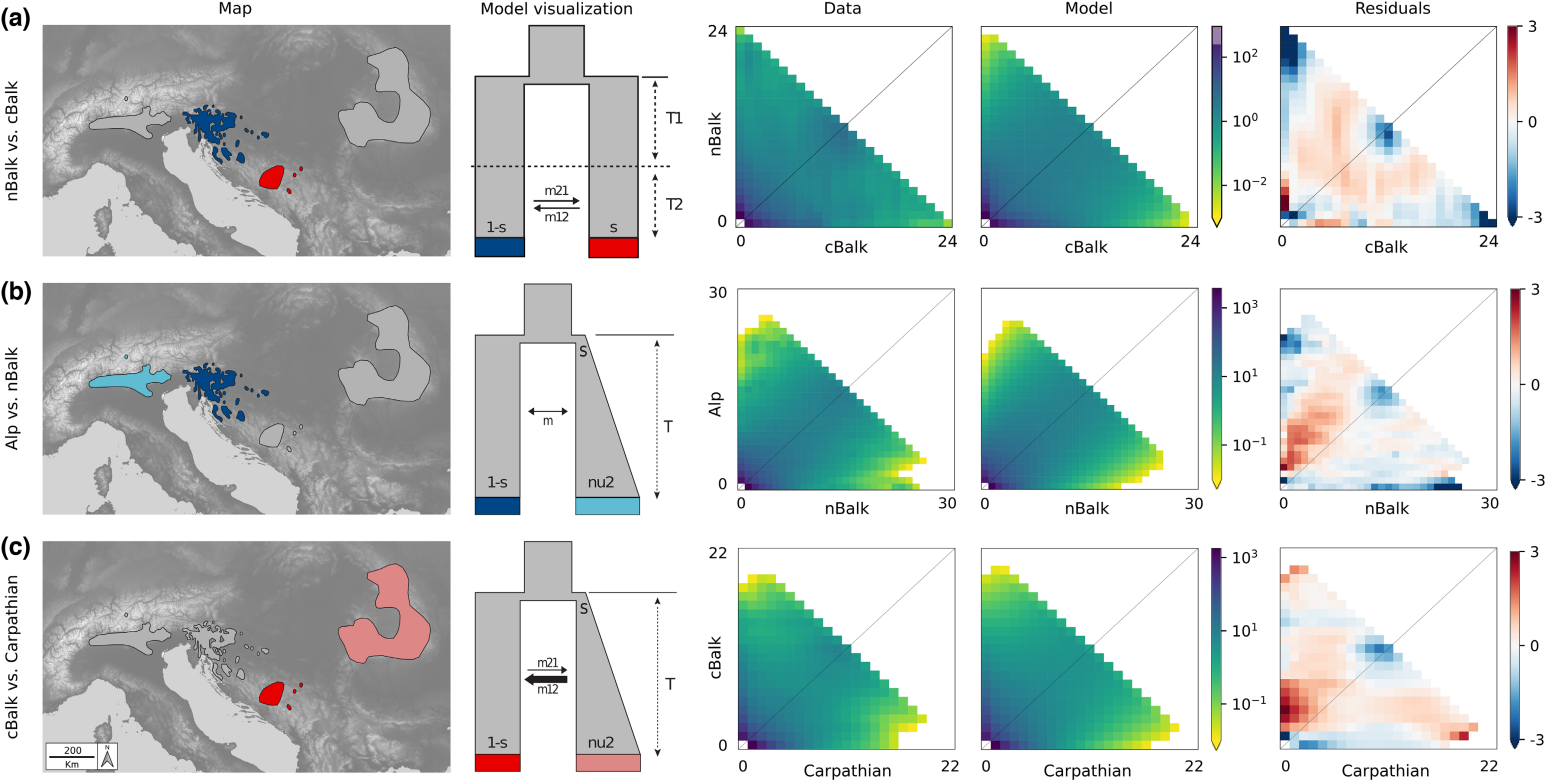
Best‐performing demographic models representing evolutionary relationships between genetic groups of *Euphorbia carniolica*. (a) *nBalk* versus *cBalk*. Vicariant divergence without gene flow and secondary contact with asymmetrical gene flow. (b) *Alp* versus *nBalk*. Founder event with exponential size growth, continuous symmetric migration (*founder_sym*). (c) *cBalk* versus *Carp*. Founder event with exponential size growth, continuous asymmetric migration (*founder_asym*; main direction of migration indicated by thickness of arrow). Maps show the population pairs captured in each model. Best‐fit models and corresponding parameters for each model are visually represented (effective population size, nu; age of split or period, T; migration rate, m; fraction of ancestral population that goes into the founding population, s; all parameter values are given in Table 1). Underlying empirical (Data) and simulated (Model) two‐dimensional joint site frequency spectra (2D‐JSFS; colours depict log‐scaled numbers of sites in each grid cell, values on *x* and *y* axes depict the number of alleles), and resulting residuals are shown as two‐dimensional histograms.

The best‐fitting models for all population pairs are shown in Figure 3, estimated parameters are given in Table 1, and results from the model testing in Table S5. Vicariance models incorporating an initial split without gene flow followed by a period with asymmetric gene flow (vic_sec_contact_asym_mig) showed the best fit when testing directionality between the groups *nBalk* and *cBalk*, independent of which group was defined as ancestral (Table S5). When comparing these best‐fitting models, the model in which *nBalk* was defined as ancestral performed better (*ω*_*i*_ = 1, Figure 3; Table S5). Model parameters suggested that the population size of *cBalk* after the initial split was smaller (*s* = 0.2871) and that gene flow was larger from *nBalk* to *cBalk* (m12 = 0.7166, m21 = 1.4306, Table 1).

**TABLE 1:** Unscaled parameter estimates (nuA, effective population size of ancestral population; nu, effective population size; m, migration; T, time; s, founding fraction; mutation parameter; Gutenkunst et al., 2009) estimated from the best‐performing demographic models (vic_sec_contact_asym_mig: split with no gene flow, followed by period of asymmetrical gene flow; founder_asym: Founder event with exponential size growth, continuous asymmetric migration; founder_sym: Founder event with exponential size growth, continuous symmetric migration).

Groups	Best model	Theta	nuA	nu1	nu2	m	m12	m21	T1	T2	T	s
*nBalk* vs. *cBalk*	vic_sec_contact _asym_mig	260.6	7.397	0.5956	1.054	–	0.717	1.430	0.467	0.139	–	0.287
*cBalk* vs. *Carp*	founder_asym	1788.7	0.269	0.1326	3.333	–	12.939	1.09	–	–	0.456	0.149
*nBalk* vs. *Alps*	founder_sym	950.9	1.857	0.8892	11.469	1.937	–	–	–	–	1.271	0.055

*Note*: Details on parameter and model definitions are given in Table S3; parameters correspond to the model illustrations in Figure 3.

Founder event scenarios (‘founder_sym’ and ‘founder_asym’) with migration were clearly the best‐fitting models within the two main groups, that is, for *nBalk* versus *Alp*, and for *cBalk* versus *Carp* (*ω*_*i*_ = 0.99 and *ω*_*i*_ = 1, Figure 3; Table S5). In both cases, an initial split of the derived population (i.e. *Alp* and *Carp*) from the ancestral population (i.e. *nBalk* and *cBalk* respectively) with subsequent exponential growth after foundation was suggested. For *nBalk* versus *Alp*, this scenario included symmetric migration (*m* = 1.93), whereas in case of *cBalk* versus *Carp*, migration was asymmetric after the founding events (*Carp* to cBalk: *m*12 = 12.93; *cBalk* to *Carp*: *m*21 = 1.09). The fraction founding the derived population was found to be larger in case of *cBalk* versus *Carp* (*s* = 0.15) compared to *nBalk* versus *Alp* (*s* = 0.05).

### 3.5 Relative genome size estimation

**FIGURE 4:**
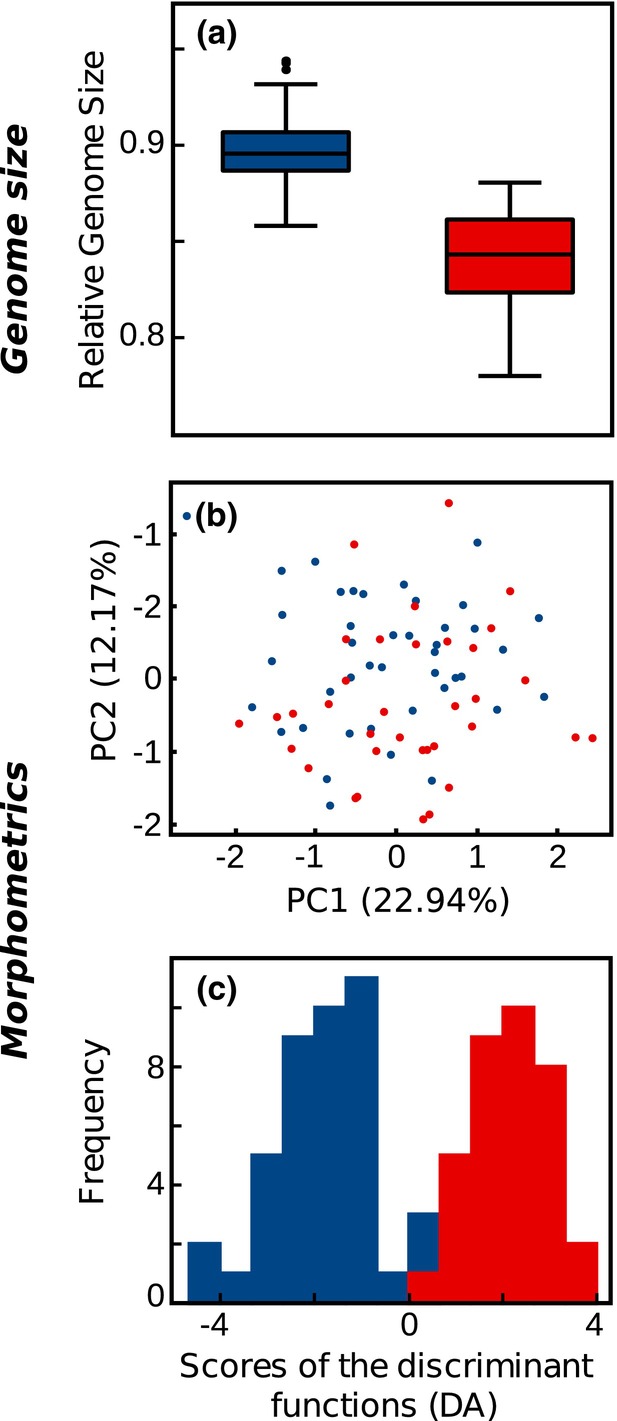
Differentiation between the two main groups within *Euphorbia carniolica* according to relative genome size and morphology (blue, *Northern Balkan–Alpine Group*; red, *Central Balkan–Carpathian Group*). (a) Boxplots of relative genome size measurements of both groups (boxes: Q1–Q3 interquartile range or IQR, centre line: median, whiskers: minima and maxima defined as Q1 − 1.5*IQR and Q3 + 1.5*IQR respectively). (b) Discriminant analysis of morphometric data. (c) Ordination of morphometric data resulting from principal component analysis.

RGS was estimated for 235 samples from 82 populations, 122 samples from 43 populations of the *Northern Balkan–Alpine Group*, and 113 samples from 39 populations of the *Central Balkan–Carpathian Group* (Data S1). The RGS ranged from 0.858 to 0.944 in the former and from 0.780 to 0.881 in the latter group (Figure 4a; Figure S7) and these differences were significant (Kruskal–Wallis *H* test: *p* < .001).

### 3.6 Morphological differentiation

Morphological character states for all characters are presented in Data S2. A PCA based on vegetative characters revealed a strong overlap in morphological space of the main genetic groups (Figure 4b). Nevertheless, 96.1% of individuals were correctly classified to the two main groups; Figure 4c shows the strong differentiation between both groups, with only a slight overlap (Wilks' Lambda = 0.21, χ^2^ = 91.58, df = 34, *p* < .001). Characters that contributed most to this separation were those describing size and form of stem, ray and raylet leaves (characters 14, 38, 39, 15, 29, 16, 20, 27 in Table S4), length of the terminal rays (11) and length of the trichomes (25).

The PCA scatter plot based on cyathium and fruit characters (Figure S8A) also showed a strong overlap in morphological space of both groups. The DA (Figure S8B) indicated a differentiation between the two groups, but with a broad overlap (Wilks' Lambda = 0.35, χ^2^ = 23.49, df = 19, *p* = .216). The characters that contributed most to the separation between the groups were describing fruit warts (57–59), fruit size (51–55) and length of the trichomes (25).

### 3.7 Modelling lineage occurrences and niche overlap

**FIGURE 5:**
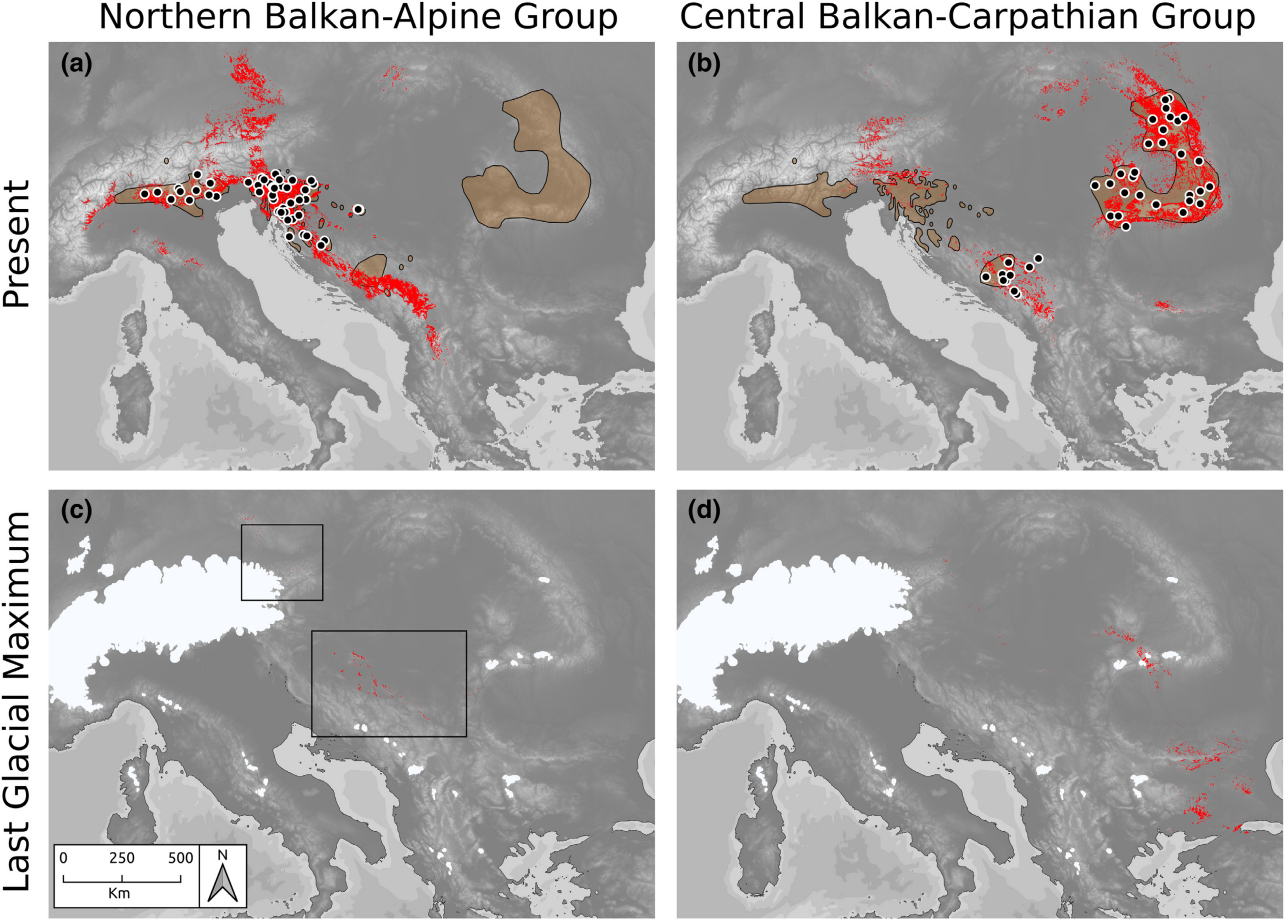
Predicted occurrences (indicated in red) of the two main genetic groups within *Euphorbia carniolica* inferred via species distribution modelling based on present‐day climatic conditions (a, b) and climatic conditions of the LGM 20 ka (c, d). Polygons represent the extant distribution range of *E*. *carniolica*, and symbols indicate populations used for modelling. Enclosing rectangles in (c) intend to enhance visibility of small occurrence patches. White areas in (c) and (d) represent ice cover under LGM conditions (Ehlers et al., 2011).

The accuracy of SDMs for both the *Central Balkan–Carpathian Group* and the *Northern Balkan–Alpine Group* was high (True Skill Statistic scores 0.945 and 0.939 respectively). Under current climatic conditions, the spatial pattern of predicted occurrences differed strongly between the two groups and largely reflected their present‐day distributions (Figure 5a,b). However, the predicted distributions of the two groups overlapped in the Eastern Alps and the central Balkan Peninsula. Retrospective SDMs indicated potential Last Glacial Maximum (LGM; 21 ka bp) refugia for the *Northern Balkan–Alpine Group* at the southwestern margin of the Bohemian Massif, in the northeastern Alps and the northeastern Balkan Peninsula (Figure 5c). Potential refugia for the *Central Balkan–Carpathian Group* were identified in the western Southern Carpathians and the Apuseni Mts., the eastern Stara Planina and the southeastern‐most Balkan Peninsula (eastern Thrace; Figure 5d).

Niches of the two main groups overlapped (Schoener's *D* = 0.31, Figure S9). Equivalency and similarity tests showed that the lineage‐specific niches were neither equivalent (*p* = .001) nor more similar than expected by chance (*p* = .089; Figure S9). Niche breadths of the *Northern Balkan–Alpine Group* and the *Central Balkan–Carpathian Group* were 0.45 and 1.51 respectively. The latter group included large fractions of the former's niche and, in addition, extended towards higher seasonality of temperature and precipitation represented by bio4 and bio15, respectively (Figure S9).

## 4 DISCUSSION

### 4.1 Mid‐Pliocene origin of *Euphorbia carniolica*

In support of the hypothesis of a pre‐Pleistocene origin of illyricoid DFUS (Trinajstić, 1992; Turrill, 1929), the divergence of *E. carniolica* from its sister *E*. *altaica* was dated to the middle Pliocene ca. 3.6 Ma (Figure 1b). It falls in a period, when (warm‐)temperate deciduous forests—that had covered large, continuous parts of Eurasia (Salzmann et al., 2008)—started to disintegrate due to gradual aridification. At the same time, grasslands started to expand in inner Eurasia (Hurka et al., 2019), posing strong barriers for forest species and thereby facilitating allopatric diversification. At the same time, climatically induced vegetation fragmentation might have caused large‐scale extinctions of forest species in previously forested areas as indicated by the large distance between extant ranges of *E. carniolica* and its sister *E. altaica*; a pattern that has been observed also in other DFUS of pre‐Pleistocene origin (Bartha et al., 2015; Sun et al., 2005; Yesson et al., 2009). Our findings complement evidence of pre‐Pleistocene origins of the DFUS *Cyclamen purpurascens* (Yesson et al., 2009), *Cardamine enneaphyllos* (L.) Crantz, *C. trifolia* L., *C. waldsteinii* Dyer (Huang et al., 2020; Ru et al., 2022), *Euphorbia dulcis* L. (this study; Figure S6) and *Knautia drymeia* (Rešetnik et al., 2016). However, the question if this pattern is pervasive for other (illyricoid) DFUS in southeastern Europe would need to be tested in a multispecies framework.

### 4.2 Early Pleistocene divergence of *E. carniolica* across a major Balkan phylogeographic break

Deep genetic divergence within *E*. *carniolica* into two groups (*Northern Balkan–Alpine Group* and *Central Balkan–Carpathian Group*) was supported by phylogenetic and clustering analyses (Figures 1 and 2), by demographic modelling that consistently inferred vicariance as the best‐fitting process behind the observed divergence (Figure 3a), by significantly different genome sizes pointing to long‐term divergent evolution (Figure 4a) and by significantly different realised niches (Figure 5). Whereas the differences in RGS between both groups are accompanied with chromosome number differentiation remains unknown, as only a single chromosome count of 2*n* = 16 has been reported for this species from Italy (Polatschek, 1971). The split (Figures 1a,b and 2a) between the two main groups occurred ca. 2.2 Ma (Figure 1b) and was most likely triggered by climatic changes in the early Pleistocene, which caused range loss and fragmentation of previously continuous forests (Leroy, 2007; Leroy et al., 2011). Before this divergence at the Pliocene/Pleistocene transition (2.6 Ma), the vegetation in Central and Southern Europe was comparable with the present one (Szabó et al., 2022), and extensive forests have likely enabled a continuous distribution of *E. carniolica*. In the same line, demographic modelling showed that an initial separation of central Balkan populations (*cBalk*) occurred via vicariance from populations in the northwestern Balkan Peninsula (*nBalk*; Figure 3a).

Despite this deep divergence, the *Central Balkan–Carpathian Group* and the *Northern Balkan–Alpine Group* are currently separated only by a narrow distribution gap (Figure 1d). Interestingly, this gap coincides with a phylogeographic break seen in many other ecologically divergent species and species groups, while the underlying causes are under debate (Đurović et al., 2021; Španiel & Rešetnik, 2022). Given the modelled climatic suitability for *E*. *carniolica* in this area (Figure 5a) and the fact that beech forests do occur there today (Magri et al., 2006), we hypothesise that the species has occurred in this gap in the past, and became extinct when forests disappeared during a cold stage. Viewed differently, our findings suggest that the present‐day distribution range of *E*. *carniolica* was co‐shaped by incomplete range filling (Svenning et al., 2008; Willner et al., 2009), which applies also to other DFUS in this area (Willner et al., 2023). The outlined scenario might have been additionally enforced by the presence of siliceous bedrock in parts of central Bosnia and Herzegovina (e.g. Vranica; Asch, 2005) within the distribution gap that could have impeded colonisation of the primarily calciphilous species such as *E*. *carniolica* or *Cyclamen purpurascens* (Slovák et al., 2012).

A scenario of periodical gene flow and isolation between the *Central Balkan–Carpathian Group* and the *Northern Balkan–Alpine Group* is corroborated by a broad admixture cline bridging the intervening gap (Figure 2a), and more explicitly, by the best‐fitting demographic model that captures vicariance with secondary asymmetric gene flow (Figure 3a; Table 1). Apart from that, the conflicting position of population BH70 in the ITS phylogeny also points to a stronger connection of the two groups in the past (Figure 1a,b).

### 4.3 Multiple LGP refugia in the Balkan Peninsula and the Carpathians

Our results corroborate the key role of the northwestern Balkan Peninsula as a major refugium and source for postglacial expansion, which is in line with traditional views on biogeography and refugial dynamics of DFUS and deciduous forests (Magri et al., 2006; Trinajstić, 1992; Turrill, 1929). However, we challenge the singularity of this refugium, and show that *E. carniolica* survived the LGP in several refugia apart from the northwestern Balkan Peninsula. Specifically, these refugia are the central Balkan Peninsula and the Carpathians, and—less likely—possibly also the margin of the western part of the Southern Limestone Alps.

We found that populations of *E*. *carniolica* (*cBalk*) have persisted in the central Balkan Peninsula since the early Pleistocene (Figure 1b), which corroborates the above‐outlined scenario of its wider distribution in the past. The long‐term persistence of a DFUS in this area also raises the question if the central Balkan refugium was in fact similarly important for deciduous trees and associated DFUS as the northwestern Balkan refugium. Phylogenetic analyses highlighting the independence and the old age of *cBalk* (Figures 1 and 2), and demographic analyses pointing to the role of *cBalk* as a source for later diversifications and expansions (Figure 3), definitely support this hypothesis. However, to finally clarify whether this important finding reflects a general pattern, data from other species are needed.

Range expansion from the central Balkan Peninsula to the Carpathians involved diffusion rather than long‐distance dispersal, as illustrated by the relatively large fraction of the ancestral *cBalk* population founding the derived *Carp* population (*s* = 0.15; Table 1; Figure 3c), as well as by several plastid haplotypes shared between both groups (Figure 1d). Divergence of *Carp* was followed by exponential population growth and strong backward migration to *cBalk* (Figure 3c; Table 1), which suggests that the intervening area between both ranges was continuously populated at some point. Hypothetical LGP forest refugia at the foothills of the Carpathians have been discussed for a long time (Gömöry et al., 2003; Magri et al., 2006; Magyari, 2002; reviewed in Mitka et al., 2014); however, we are the first to present genomic evidence for LGP survival of a DFUS in this area, which we interpret as a proxy for the existence of forest.

More specifically, our results corroborate the existence of two Carpathian refugia for DFUS, situated in the eastern part of the Southern Carpathians and in the Eastern Carpathians, where we found the divergent genetic groups *esCarp* and *eCarp* (Figure 2b,c). Within the Carpathians, the phylogeographic structure (Figure 2c) clearly reflects patterns observed in other, yet mostly subalpine and alpine species such as *Campanula alpina* Jacq. (Campanulaceae), *Hypochaeris uniflora* Vill. (Asteraceae) or *Onobrychis transsilvanica* Simonk. (Fabaceae), suggesting that historical processes have influenced plants with different ecological adaptations in a similar manner (Mráz & Ronikier, 2016; Ronikier, 2011).

In contrast to the evidence supporting the existence of refugia in the Balkan Peninsula and the Carpathians, evidence for a refugium in the western part of the Southern Limestone Alps is inconclusive. The Euganean Hills, which are located close to the southern margin of the Alps, have been suggested as refugium for deciduous trees (Gubler et al., 2018; Kaltenrieder et al., 2009). Molecular dating (Figure 1b), but also the relative timing of the splits (Figure 2a; Table 1), suggests that the divergence of *Alp* might be younger than that of *Carp*. Most of the employed analyses failed to delimit *Alp* as a distinct group (Figure 1; Figure 2b,c), and only phylogenetic analyses based on RADseq resolved a well‐supported monophyletic group *Alp* (BS > 75%) nested within the *Northern Balkan–Alpine Group* (Figure 2a). We interpret this finding as the result of a recent—probably Holocene—founder event and subsequent genetic drift, a scenario that was also supported by demographic modelling (Figure 3b). Contrary to the scenario suggested for *cBalk* and *Carp*, *Alp* was founded by a very small fraction (*s* = 0.05, Table 1) of the ancestral *nBalk* populations, suggesting a strong bottleneck. Furthermore, *Alp* lacks a clear internal structure (Figure 2), which is in line with a recent founder event. The northwesternmost populations of *nBalk* likely acted as source, as they also share their plastid haplotype with the populations of *Alp* (Figure 1d). Population foundation was followed by exponential growth and likely by spatial range expansion (Figure 3b).

### 4.4 Limited congruence between SDMs and genomically inferred LGP refugia

Refugia that were well supported by genomic evidence are not corresponding well with SDM predictions for *E*. *carniolica*. SDMs failed to infer suitable areas in refugia in the northwestern‐most Balkan Peninsula and the adjacent Southern Limestone Alps, and only small and disjoint suitable areas in the southwestern and southern periphery of the Pannonian Basin were resolved (Figure 5). In contrast, regions north of the Alps were modelled as suitable for both current and LGM conditions, while the species is not occurring there today. The absence of the species north of the Alps might be a consequence of dispersal limitation, as the Alps likely prevented northward dispersal (Willner et al., 2023). We suspect that this mismatch of SDMs and genomic data stems from the combination of four factors. (i) Microrefugia with locally suitable conditions could sustain populations even in areas that were predicted to be unsuitable because of poor resolution of climatic data (Stark & Fridley, 2022). (ii) SDMs predict potential suitability of an area as a continuous metric, which has been transformed into binary presence/absence predictions using a threshold (Supplementary Material; Figure 5). Modelled suitabilities for LGM conditions were generally low, and suitable areas were small and disjoint, which fits the assumption that macroclimatic conditions in the LGP were generally unsuitable for forest species. However, predicted suitabilities in the northwestern Balkan Peninsula were only slightly below the presence/absence threshold (Figure S10). This may indicate that differences in suitability between areas actually acting as microrefugia and surrounding unsuitable areas might have been only marginal. (iii) The niche of *E. carniolica* might have evolved since the LGM, and its past niche might thus be outside the species' present day niche. Although the prevalence and strength of such relatively recent niche shifts have rarely been studied, multispecies data (Razgour et al., 2019) and models (Cotto et al., 2017) showed that climatic niches can evolve rapidly over short time periods, such as the period after the LGM. Finally, (iv) some past climates likely have no present‐day representation in the species' range. As a consequence, the environmental niche fitted with the SDMs might be too narrow. This fits results of a recent study using a dynamic modelling approach (i.e. combining SDM predictions with modelling of the local demography and dispersal of species), which found that a niche breadth extension of 20%, strong dispersal limitation and occasional long‐distance dispersals were necessary to realistically model the expansion of five DFUS, among them *E. carniolica*, from their potential LGM refugia (Willner et al., 2023). Thus, a static approach such as climate‐based retrospective SDMs may be an oversimplification that finally fails to identify refugia, as species may indeed have wider ecological amplitudes than their current distribution ranges suggest.

### 4.5 Morphological stasis is consistent with weak ecological differentiation in *E. carniolica*

Ecological differentiation between the *Central Balkan–Carpathian Group* and the *Northern Balkan–Alpine Group* was found to be significant but relatively weak, despite the deep genetic divergence (Figure 5a,b; Figure S9). Specifically, the current climatic niche of the *Central Balkan–Carpathian Group* is much broader and extends towards higher seasonality of temperatures and precipitation, reflecting the larger continentality of eastern European climate, and largely includes the niche of the *Northern Balkan–Alpine Group*.

Consistent with the overlapping ecological niches, the morphological divergence between the two groups is shallow. A strong overlap in morphological characters was observed between groups in the PCA (Figure 4b), even if the slight quantitative differences across characters led to separation of the groups in the DA (Figure 4c). This is consistent with general observations of morphological stasis (i.e. slow rates of morphological change) in forest species (Milne & Abbott, 2002; Parks & Wendel, 1990; Wen, 2001; Williamson, 1987). Morphological stasis has been observed even between populations of ‘Tertiary’ relict forest species that today occur on different continents (Milne & Abbott, 2002; Wen, 2001). It might reflect similar climatic tolerances of widely disjunct populations (Tiffney & Manchester, 2001), as similar habitat and climatic conditions that have remained stable over long periods can foster stabilising selection (Wen, 2001). The absence of clear morphological differentiation despite pre‐Pleistocene divergence has recently been found also between European and Asian populations of *E. amygdaloides* L., a typical forest species that often co‐occurs with *E. carniolica* (Pahlevani & Frajman, 2023). In line with the weak morphological and ecological divergence within *E. carniolica* and overlapping—albeit significantly different—RGS, accompanied by admixture between the two allopatric RADseq groups, we consider the *Central Balkan–Carpathian Group* and the *Northern Balkan–Alpine Group* as two subspecies and provide a taxonomic treatment including descriptions and typifications in Appendix 1.

## 5 CONCLUSIONS

We have shown that the spatiotemporal diversification of forest species may be complex. Instead of a single cold‐stage refugium in the northwestern Balkan Peninsula, we identified several refugia for *E*. *carniolica*, challenging the oversimplified but still widespread view that large, homogenous refugia for temperate forest species existed in southern Europe (Hewitt, 1999). Given that *E. carniolica* is tightly linked to deciduous forests, we stress that the identified LGP refugia are useful proxies for refugia of deciduous forests, and thus aid in reconstructing the history of this forest type in Europe. From a biogeographic point of view, it is particularly intriguing that *E*. *carniolica* and *E. altaica* are sister species despite a disjunction of several thousand kilometres spanning across presently forest‐free inner Asia, which might hint at a pre‐Pleistocene connection under a warmer, more humid climate. While such disjunctions have been highlighted (but never explicitly studied) in temperate forest species in the past, future studies elucidating the processes conferring these disjunctions are urgently needed to help unravelling the pre‐Pleistocene evolution of temperate forest species in Eurasia and to provide a fresh perspective on Eurasian forest biogeography in general.

## AUTHOR CONTRIBUTIONS

PK, EZ, BF, WW, KH and PS conceived the study. PK, BF and PS co‐wrote major parts of the manuscript. PK and EZ analysed genomic data and did demographic modelling analyses. BF analysed morphometric data, single gene data and performed molecular dating analyses. KH, JW and WW did all analyses related to species distribution modelling, wrote corresponding sections in the manuscript and significantly contributed to manuscript writing at a later stage. All authors read and edited the final version of the manuscript.

## FUNDING INFORMATION

This work was financed by the Austrian Science Fund (FWF, project P29413 ‘Range formation of beech forest understory herbs’ to PS). Research support of EZ was provided as part of a long‐term research project of the Czech Academy of Sciences, Institute of Botany (RVO 67985939).

## CONFLICT OF INTEREST STATEMENT

The authors declare no conflicts of interest.

## Supporting information


Appendix S1

Data S1

Data S2

## Data Availability

Single gene sequence data and RADseq reads have been made available via NCBI's Genbank (JN009933, JN010033, OQ519866–OQ539523) and Short Read Archive (BioProject PRJNA983383, accession nos SAMN35724938–SAMN35725141), respectively (details in Data S1). Locality data and detailed collection data, as well as relative genome size measurements are provided in tabular format (Data S1). Morphometric data are provided in Data S2.

## APPENDIX 1

### TAXONOMIC TREATMENT

Based on phylogenetic divergence, genome size differentiation and allopatric distribution, despite minor morphological differences, we recently described new species in the *E. nicaeensis* All. (Stojilkovič et al., 2022) and *E. amygdaloides* alliances (Pahlevani & Frajman, 2023), and resurrected neglected species treated as synonyms in the *E. verrucosa* group (Caković et al., 2021), that both diversified in the Pleistocene. On the contrary, weak genetic differentiation not clearly following the morpho‐taxonomic boundaries led to the lumping of two species into a single, morphologically and ecologically variable species, and rather recognition of two subspecies with overlapping ranges in *E. spinosa* (Stevanoski et al., 2020), or to the recognition of a single, morphologically and ecologically variable species in *E. villosa*, where the previously recognised taxa appear to be merely ecotypes (Frajman et al., 2016). Given the deep phylogenetic divergence, genome size differentiation and allopatric distribution of the two main genetic groups within *E. carniolica*, their recognition as independent species could be justified, but strong signals of admixture as well as strongly overlapping morphology render recognition of two subspecies more suitable. The *Northern Balkan–Alpine Group* corresponds to the type subspecies, whereas we apply the existing name *Euphorbia carniolica* subsp. *expansa* (Janka) Nyman for the *Central Balkan–Carpathian Group*.

#### Identification key

Metric values presented in the key and the descriptions correspond to the 10 and 90 percentiles, supplemented by extreme values in parentheses.

**1.** Stems glabrous or pubescent, with 0–50(90) trichomes on 1 cm × 1 mm stem surface. Middle stem leaves (28)36–61(68) mm long, (2.0)2.2–3.4(5.1) times longer than wide, tip angle (53)64–104(109)°. Raylet leaves widest at 0.4–0.6(0.7) of their length. Cyathial involucre (0.6)1.6–2.4(2.6) mm long. Capsules (0.8)1.3–4.2(5.2) × (0.8)1.2–4.4(5.0) mm. Seeds 3.0–3.4(3.9) × (1.7)2.1–2.5 mm. Southern Alps, northwestern Balkan Peninsula ***E*. *carniolica* subsp. *carniolica***

**1*.** Stems glabrous or pubescent with 0–110(155) trichomes on 1 cm × 1 mm stem surface. Middle stem leaves (33)41–65(79) mm long, (2.0)2.3–3.8(4.2) times longer than wide, tip angle (74)76–111(125)°. Raylet leaves widest at 0.3–0.5(0.6) of their length. Cyathial involucre (0.8)1.7–2.7(3.0) mm long. Capsules (0.8)1.4–4.5(4.8) × (0.6)1.5–3.3(5.9) mm. Seeds 2.5–3.3(3.4) × 2.0–2.4 mm. Carpathians, central Balkan Peninsula ***E*. *carniolica* subsp. *expansa***

***E. carniolica* Jacq.**, Fl. Austriac. 5: 34 (1778). ≡ *Tithymalus carniolicus* (Jacq.) Raf. in Fl. Tellur. 4: 115 (1838). –Type: [icon] Tab. XIV in Fl. Austriac. 5 (1778), lectotype designated here. Locality indicated in the protologue: [Slovenia] ‘Crescit circa Idriam’. Note: as we were not able to trace any herbarium specimens that could be considered original material, we designate the iconography of the species published by Jacquin (1778) as a part of the protologue.

***E. carniolica* subsp. *carniolica***

≡ *Euphorbia ambigua* Waldst. & Kit. ex Willd. in Sp. Pl. 2, ed. 4: 910 (1799). –Type: [Croatia] ‘In sylvis ad Koreniczam [Korenica], majus specimen in valle, per quem e Merszin ad Vrelo descenditur’ Herbar. Kitaibel no. 4174. (BP XIV/55 – photo!), **lectotype**, designated here. Additional original material: [Romania] ‘Pl. rar. Hung. Bane (?) Rezbanya [Băița] in sylva’. Herbar. Kitaibel no. 4173.BP XIV/54. (https://gallery.hungaricana.hu/hu/search/results/?list=eyJmaWx0ZXJzIjogeyJTT1VSQ0UiOiBbIk1VWkVfT1JTWl9UVFVEIl19LCAicXVlcnkiOiAiRXVwaG9yYmlhIGFtYmlndWEifQ)

Note: *Euphorbia mehadiensis* Kit. was often listed as a synonym for *E. carniolica* (e.g. Govaerts et al., 2000), but the type actually corresponds to *Euphorbia epithymoides* L.

*Description*: Glabrous or pubescent perennial, with stems (18)24–48(57) cm high and (1.5)1.9–3.1(4.5) mm thick, arising from a woody stock. Middle stem leaves obovate‐oblong, obtuse or cuspidate, (28)36–61(68) × (9)12–23(30) mm, (2.0)2.2–3.4(5.1) times longer than wide, widest at 0.5–0.7 of the length, with an attenuate to rounded basis and an acute to rounded apex with the tip angle (53)64–104(109)°. Leaves hairy, with 0–6(9) hairs in 1 mm^2^ at the upper leaf surface and 4–17(20) in 1 mm^2^ lower leaf surface. Terminal rays three to five, (1.6)2.7–11.3(14.2) cm long, 1–2 times dichotomous. Ray leaves (28)31–53(64) × (9)14–24(31) mm, (1.7)2.0–2.8 (3.6) times longer than wide, widest at (0.4)0.5–0.6 of the length, with mostly rounded basis and a broadly acute to obtuse apex, with the tip angle (53)60–102(124)°. Raylet leaves ovate–lanceolate, (14)19–37(49) × (4)10–21(28) mm, (1.4)1.5–2.2(3.1) times longer than wide, widest at 0.4–0.6(0.7) of the length, with rounded basis and broadly acute to obtuse apex with the tip angle (52)64–101(116)°. Cyathial involucre campanulate, (0.6)1.6–2.4(2.6) × (1.0)1.3–2.1(2.5) mm, (0.7)0.9–1.4(2.0) times longer than wide, with yellow to brownish, elliptic glands, (0.2)0.3–0.8(1.0) × 0.3–1.0(1.3) mm, (0.3)0.4–2.2(2.7) times longer than wide. Capsule glabrous, (0.8)1.3–4.2(5.2) × (0.8)1.2–4.4(5.0) mm, (0.8)0.9–1.3(2.0) times longer than wide, widest at (0.3)0.4–0.6(0.7) of the length, with warts 0.1–0.5(0.7) × 0.1–0.3(0.6) mm, (0.7)0.9–1.5(2.0) longer than wide, widest at (0.3)0.4–0.6 of the length. Styles (0.9)1.1–1.6(1.7) mm long. Seeds ovoid, smooth, brown, 3.0–3.4(3.9) × (1.7)2.1–2.5 mm, 1.2–1.7(1.8) times longer than wide, widest at 0.4–0.5 of the length. Caruncle kidney‐shaped or semi‐hemispherical, (0.3)0.4–0.6(0.9) × 0.4–0.6(0.9) mm, (0.4)0.5–1.0(2.1) times longer than wide and widest at (0.2)0.3–0.5(0.6) of the length.

*Distribution*: Southern Limestone Alps and southerly adjacent areas, northwestern Balkan Peninsula, southern Pannonian Plain (Austria, Italy, Slovenia, Croatia, Bosnia and Herzegovina).

*Habitat*: Mostly deciduous forests, particularly those dominated by beech, rarely mixed forests, mostly below timberline.

***E. carniolica* subsp. *expansa* (Janka) Nyman**, Consp. Fl. Eur. 649 (1881) ≡ *Euphorbia expansa* Janka in Linnaea 30: 600 (1860). – Type locality indicated in the protologue: [Romania] ‘In Transsilvaniae septemtrionalis silvis pr. pagum Gánts’. Type: ‘*Euphorbia carniolica*? Jacq./Gánts cserékbe és fásokba’ [Gáncs cserjésekben és fásokban = in scrubs and woodlands of Gáncs, Dumbrăveni], *leg. Czetz, IV–V [1]855* (CJ 25277 – photo!), **lectotype, designated here**.

*Description*: Glabrous or pubescent perennial, with stems (19)22–49(55) cm high and (1.6)1.9–4.0(5.5) mm thick, arising from a woody stock. Middle stem leaves obovate–oblong, obtuse or cuspidate, (33)41–65(79) × (10)13–24(29) mm, (2.0)2.3–3.8(4.2) times longer than wide, widest at (0.4)0.5–0.7 of the length, with an attenuate to rounded basis and an acute to rounded apex, with the tip angle (74)76–111(125)°. Leaves glabrous or hairy, with 0–6(9) hairs in 1 mm^2^ at the upper leaf surface and 0–14(25) in 1 mm^2^ lower leaf surface. Terminal rays three to five, (2.3)3.5–10.7(12.6) cm long, 1–2 times dichotomous. Ray leaves (30)32–548(66) × (11.3)14.5–24.9(33.1) mm, (1.7)2.0–3.0(3.2) times longer than wide, widest at (0.3)0.4–0.6 of the length, with mostly rounded basis and a broadly acute to obtuse apex with the tip angle (60)70–105(120)°. Raylet leaves ovate–lanceolate, (13)20–37(43) × (6)12–22(30) mm, (1.3)1.4–2.2(2.5) times longer than wide, widest at 0.3–0.5(0.6) of the length, with rounded basis and broadly acute to obtuse apex with the tip angle (66)77–111(130)°. Cyathial involucre campanulate, (0.8)1.7–2.7(3.0) × (1.0)1.1–2.4(2.5) mm, (0.8)0.9–1.3(1.6) times longer than wide, with yellow to brownish, elliptic glands 0.5–0.9(1.5) × (0.1)0.2–0.9(1.0) mm, 0.6–3.5(6.3) times longer than wide. Capsule glabrous, (0.8)1.4–4.5(4.8) × (0.6)1.5–3.3(5.9) mm, (0.8)0.9–1.4 times longer than wide, widest at (0.4)0.5–0.6 of the length, with warts 0.2–0.5(0.6) × 0.1–0.3(0.4) mm, (1.1)1.3–1.6(2.9) times longer than wide, widest at (0.2)0.3–0.5(0.6) of the length. Styles (1.0)1.1–1.2(1.5) mm long. Seeds ovoid, smooth, brown, 2.5–3.3(3.4) × 2.0–2.4 mm, (1.2)1.3–1.4 times longer than wide, widest at 0.4 of the length. Caruncle kidney‐shaped or semi‐hemispherical, 0.4–0.5 × 0.6–0.7 mm, 0.7 times longer than wide and widest at 0.4–0.5 of the length.

*Distribution*: Central parts of the western Balkan Peninsula, Southern and Eastern Carpathians, Apuseni Mountains (Bosnia and Herzegovina, Montenegro, Serbia, Romania, Ukraine).

*Habitat*: Mostly deciduous forests, particularly those dominated by beech, mixed forests, subalpine grasslands.
