# Supplementary material for: Evolutionary dynamics of Euphorbia carniolica suggest a complex Plio–Pleistocene history of understorey species of deciduous forest in southeastern Europe
Source: Mol Ecol. 2023 Aug 26;32(19):5350–68. doi: 10.1111/mec.17102 (PMC10946815; doi:10.1111/mec.17102)
Supplement: Supplementary file 1 — Appendix S1 [file MEC-32-5350-s003.docx]

**SUPPLEMENTARY MATERIAL**

**Evolutionary dynamics of *Euphorbia* *carniolica* suggest a complex Plio-Pleistocene history of understorey species of deciduous forest in southeastern Europe**

Philipp Kirschner^1^, Eliška Záveská^1,2^, Karl Hülber^3^, Johannes Wessely^3^, Wolfgang Willner^3^, Peter Schönswetter^1^*, Božo Frajman^1^*

^1^Department of Botany, University of Innsbruck, Innsbruck, Austria
^2^Institute of Botany of the Czech Academy of Sciences, Průhonice, Czechia
^3^Department of Botany and Biodiversity Research, University of Vienna, Vienna, Austria

*shared last authors

**Material and Methods**

**Bioclimatic variables and modelling lineage occurrences**

Data on historical and current climates were retrieved from the Chelsa Climate database (Karger et al., 2021; available at <http://chelsa-climate.org/>) with a spatial resolution of 30″. Four bioclimatic variables representing measures of the absolute values and the variation of temperature and precipitation were used: mean annual temperature (bio1), temperature seasonality (bio4), annual precipitation sum (bio12) and precipitation seasonality (bio15). These variables were checked for autocorrelation (Pearson’s r< 0.6), and in a further step, they were projected to a grid of 1×1 km cell size using the nearest neighbour method. For model projection to climatic conditions of the Last Glacial Maximum (LGM, 21 ka bp) the Community Climate System Model Version 4 was used (CCSM4; Gent et al., 2011). This model was selected as it has proven to outperform other global circulation models in Europe (Fordham et al., 2017). As additional environmental variable, we used topographical roughness defined as the standard deviation of the elevations of all 100×100 m cells (https://www.eea.europa.eu/data-and-maps/data/copernicus-land-monitoring-service-eu-dem) within each 1×1 km cell.

**Parameterization of SDMs**

For each lineage, 10000 pseudo-absences were randomly drawn from a 250 km buffered rectangle around the species presences. This selection of pseudo-absences was repeated five times. SDMs were parameterized within the BIOMOD framework version 2 (Thuiller et al., 2009) by means of four modelling techniques using their default settings: Generalised Linear Models (GLM), Generalised Additive Models (GAM), Boosted Regression Trees (GBM) and Random Forests (RF). Model runs were replicated three times (for each set of pseudo-absences) using 80% of data for model parameterization and the remaining 20% for model evaluation using the True Skill Statistic score (TSS; Allouche et al., 2006). Based on the resulting 60 parameterized models (5 pseudo-absence drawings × 3 replicates × 4 modelling techniques), we calculated ensemble projections of potential species ranges under current and LGM (i.e., 21 ka bp) climatic conditions as weighted (by the TSS value of single models) mean of the projected occurrence probabilities of the single models (Supplementary Table 10). These probabilistic ensemble projections were translated into binary predictions (occurring/missing) using the threshold that maximizes the TSS value (Liu et al., 2005).

**Analyses of climatic niches**

Niche overlap between the two main lineages of *E. carniolica*, i.e. the *Northern Balkan-Alpine Group* and the *Central Balkan-Carpathian Group*, was computed following Broennimann et al. (2012). This comprises i) calculation of two-dimensional density distributions (dd) of occurrences for each lineage; ii) determination of niche overlap in this environmental space; and iii) tests of niche identity and niche similarity. First, dd were derived from a PCA based on values of the five environmental variables (standardized to zero mean and unit variance) used for the SDM (including all cells covered by the current range of *E. carniolica* plus a buffer of 250 km). Maximum and minimum scores of the first and second axis of the PCA were used to define boundaries of the environmental space, which was divided into 100 bins along each axis resulting in a grid of 10,000 cells each, representing a unique combination of environmental variables. The dd of occurrence points of each lineage was calculated using the function *ecospat.grid.clim.dyn* applying the *adehabit* kernel smoother. Assuming that lineages of *E. carniolica* are fully allopatric, they experienced different suites of environmental conditions. To correct for this differential availability of environments, occurrence densities were divided by the dd of the environmental conditions available in the range of each lineage (buffered by 250 km). Niche overlap between the two lineages was calculated based on these corrected dd using the function *ecospat.niche.overlap*.

To test if the two lineages of *E*. *carniolica* are effectively identical in their realised distributions in environmental space, an ‘equivalency test‘ (Warren et al., 2008) was done. In this test, a niche overlap metric (Schöner´s D; Schoener, 1970) is computed for the empirical occurrences within each group. In addition, a null distribution of the same metric was generated by repeatedly randomising group identity of each occurrence while keeping the sample sizes of the empirical data. Comparing the empirical value against the null distribution tests the null hypothesis of identical niches at a given level of confidence (here alpha=0.05; Warren et al., 2008). In the additionally used ‘similarity test’ (Warren et al., 2008) a null distribution is generated by randomly shifting the observed dd of occurrences of one lineage in environmental space and comparing it to the empirical distribution of the other lineage, i.e. the centre of the simulated dd is picked randomly among available environments ([Broennimann et al., 2012](https://www.zotero.org/google-docs/?broken=0Mx99V)). Rejecting the null hypothesis of this test indicates that lineages are more similar in their environmental distributions than expected by chance given their respective ranges (Warren et al., 2021). Both tests were done using 100 repetitions using the functions *ecospat.niche.equivalency.test* and *ecospat.niche.similarity.test* (both included in *ecospat;* Di Cola et al., 2017).

**BIBLIOGRAPHY**

Allouche, O., Tsoar, A., & Kadmon, R. (2006). Assessing the accuracy of species distribution models: Prevalence, kappa and the true skill statistic (TSS). *Journal of Applied Ecology*, *43*(6), 1223–1232. https://doi.org/10.1111/j.1365-2664.2006.01214.x

Broennimann, O., Fitzpatrick, M. C., Pearman, P. B., Petitpierre, B., Pellissier, L., Yoccoz, N. G., Thuiller, W., Fortin, M.-J., Randin, C., Zimmermann, N. E., Graham, C. H., & Guisan, A. (2012). Measuring ecological niche overlap from occurrence and spatial environmental data. *Global Ecology and Biogeography*, *21*(4), 481–497. https://doi.org/10.1111/j.1466-8238.2011.00698.x

Charles, K. L., Bell, R. C., Blackburn, D. C., Burger, M., Fujita, M. K., Gvoždík, V., Jongsma, G. F. M., Kouete, M. T., Leaché, A. D., & Portik, D. M. (2018). Sky, sea, and forest islands: Diversification in the African leaf-folding frog *Afrixalus paradorsalis* (Anura: Hyperoliidae) of the Lower Guineo-Congolian rain forest. *Journal of Biogeography*, *45*(8), 1781–1794. https://doi.org/10.1111/jbi.13365

Di Cola, V., Broennimann, O., Petitpierre, B., Breiner, F. T., D’Amen, M., Randin, C., Engler, R., Pottier, J., Pio, D., Dubuis, A., Pellissier, L., Mateo, R. G., Hordijk, W., Salamin, N., & Guisan, A. (2017). ecospat: An R package to support spatial analyses and modeling of species niches and distributions. *Ecography*, *40*(6), 774–787. https://doi.org/10.1111/ecog.02671

Fordham, D. A., Saltré, F., Haythorne, S., Wigley, T. M. L., Otto-Bliesner, B. L., Chan, K. C., & Brook, B. W. (2017). PaleoView: A tool for generating continuous climate projections spanning the last 21 000 years at regional and global scales. *Ecography*, *40*(11), 1348–1358. https://doi.org/10.1111/ecog.03031

Gent, P. R., Danabasoglu, G., Donner, L. J., Holland, M. M., Hunke, E. C., Jayne, S. R., Lawrence, D. M., Neale, R. B., Rasch, P. J., Vertenstein, M., Worley, P. H., Yang, Z.-L., & Zhang, M. (2011). The Community Climate System Model Version 4. *Journal of Climate*, *24*(19), 4973–4991. https://doi.org/10.1175/2011JCLI4083.1

Karger, D. N., Nobis, M. P., Normand, S., Graham, C. H., & Zimmermann, N. E. (2021). CHELSA-TraCE21k v1.0. Downscaled transient temperature and precipitation data since the last glacial maximum. *Climate of the Past Discussions*, 1–27. https://doi.org/10.5194/cp-2021-30

Liu, C., Berry, P. M., Dawson, T. P., & Pearson, R. G. (2005). Selecting thresholds of occurrence in the prediction of species distributions. *Ecography*, *28*(3), 385–393. https://doi.org/10.1111/j.0906-7590.2005.03957.x

Schoener, T. W. (1970). Nonsynchronous Spatial Overlap of Lizards in Patchy Habitats. *Ecology*, *51*(3), 408–418. https://doi.org/10.2307/1935376

Thuiller, W., Lafourcade, B., Engler, R., & Araújo, M. B. (2009). BIOMOD – a platform for ensemble forecasting of species distributions. *Ecography*, *32*(3), 369–373. https://doi.org/10.1111/j.1600-0587.2008.05742.x

Warren, D. L., Glor, R. E., & Turelli, M. (2008). Environmental niche equivalency versus conservatism: Quantitative approaches to niche evolution. *Evolution*, *62*(11), 2868–2883. https://doi.org/10.1111/j.1558-5646.2008.00482.x

Warren, D. L., Matzke, N. J., Cardillo, M., Baumgartner, J. B., Beaumont, L. J., Turelli, M., Glor, R. E., Huron, N. A., Simões, M., Iglesias, T. L., Piquet, J. C., & Dinnage, R. (2021). ENMTools 1.0: An R package for comparative ecological biogeography. *Ecography*, *44*(4), 504–511. https://doi.org/10.1111/ecog.05485

Záveská, E., Kirschner, P., Frajman, B., Wessely, J., Willner, W., Gattringer, A., Hülber, K., Lazić, D., Dobeš, C., & Schönswetter, P. (2021). Evidence for Glacial Refugia of the Forest Understorey Species *Helleborus niger* (Ranunculaceae) in the Southern as Well as in the Northern Limestone Alps. *Frontiers in Plant Science*, *12*, 839. https://doi.org/10.3389/fpls.2021.683043

**Supplementary Figures**


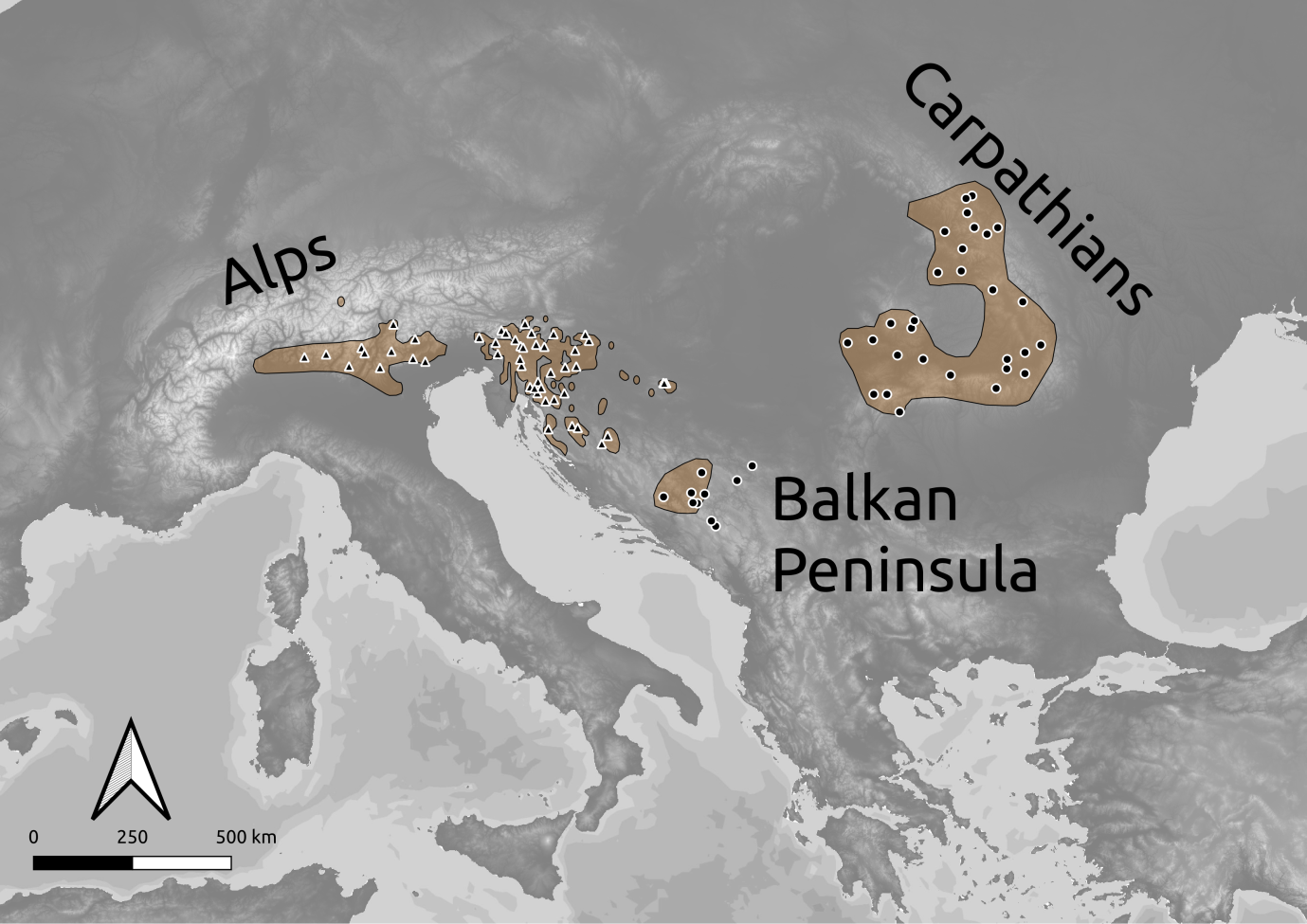
**Supplementary Figure 1.** Location of all populations of *E*. *carniolica* sampled for this study. Black dots, populations for which restriction site associated DNA data were generated; black and white dots, populations used for species distribution modelling.

**
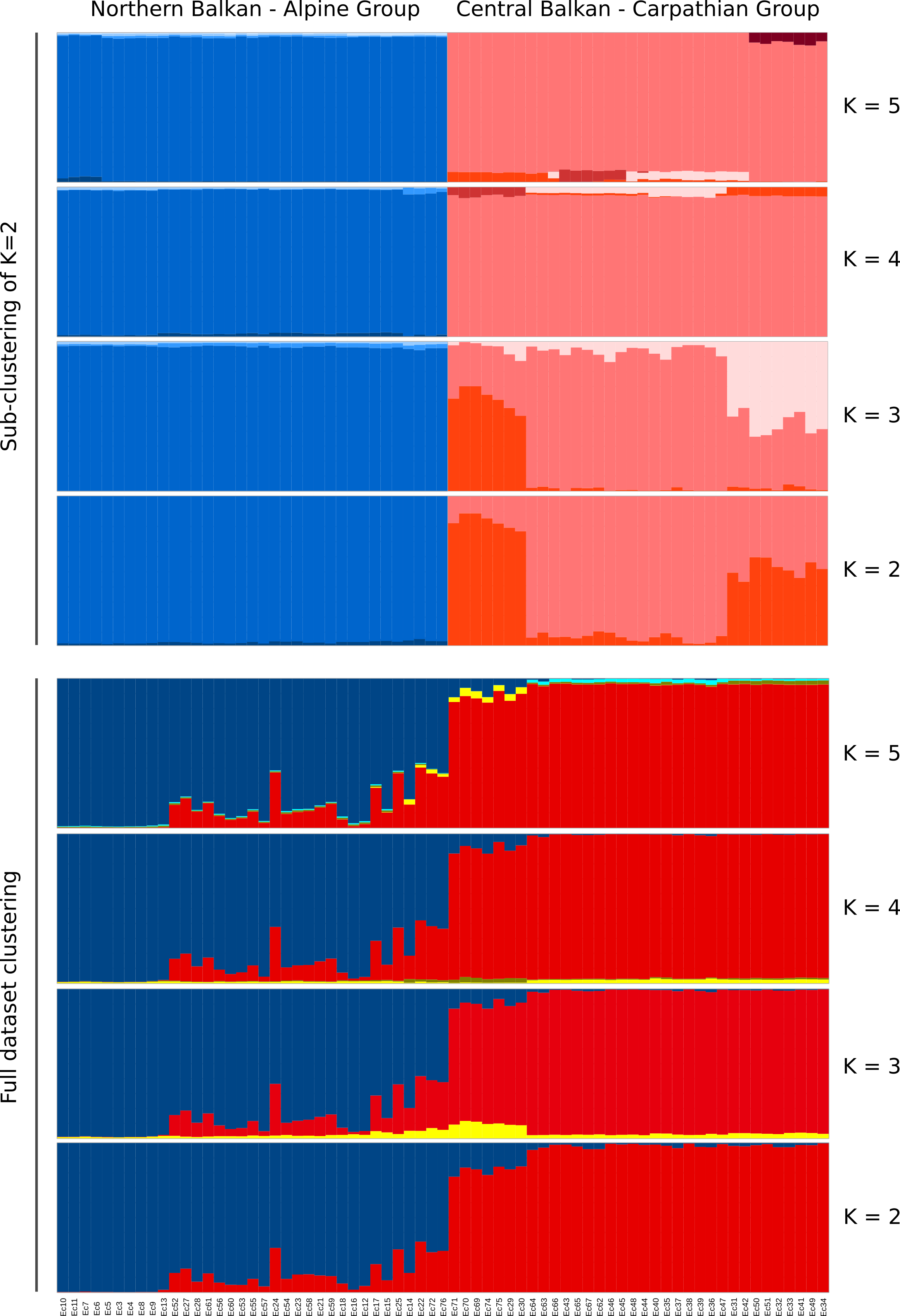
**

**Supplementary Figure 2.** Proportion of shared genetic variation resulting from Bayesian clustering analyses for K=2 to K=5. Bellow: complete dataset. Above: group wise sub-clustering within the main genetic groups, i.e. the *Northern Balkan-Alpine Group* and the *Central Balkan-Carpathian Group* inferred at K=2 of the analyses of the complete dataset. Each bar represents one population. Population numbers on the x-axis correspond to Supplementary Data 1.


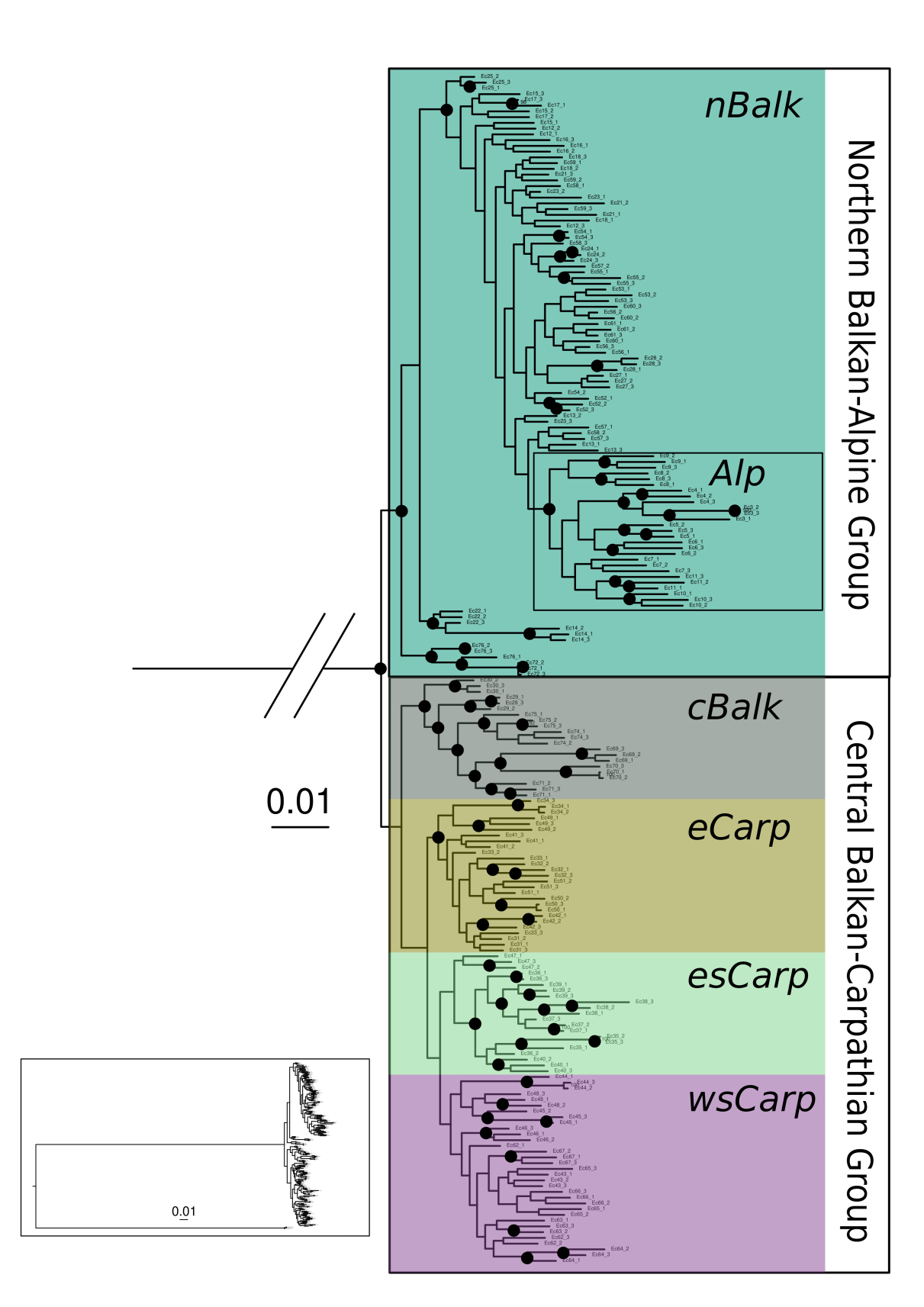


**Supplementary Figure 3.** Phylogenetic tree inferred under maximum likelihood using restriction site-associated DNA sequencing data. Genetic groups used throughout the manuscript are indicated. Black dots indicate nodes for which bootstrap support was larger than 75%. Population numbers on correspond to Supplementary Data 1. To enhance readability, the large Tree comprises only the ingroup, and the full tree including the outgroup *E*. *angulata* is given in the small caption.


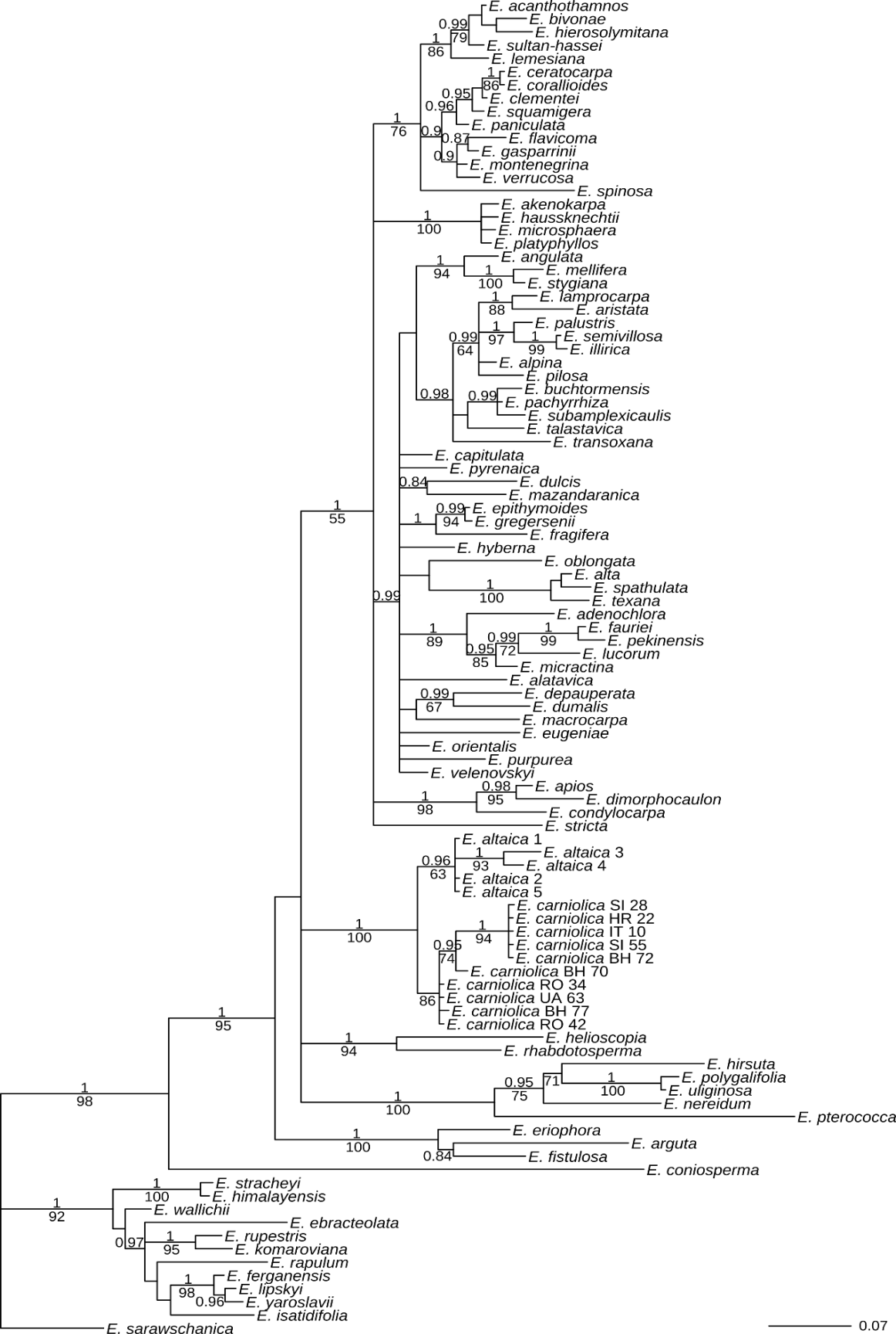


**Supplementary Figure 4.** Bayesian consensus phylogram inferred from ITS sequences showing phylogenetic position of *Euphorbia carniolica* and its sister *E. altaica* within *E.* sect. *Helioscopia*. Numbers above branches are posterior probabilities > 0.8, those below branches maximum parsimony bootstrap values > 60%. Population numbers correspond to Supplementary Data 1.


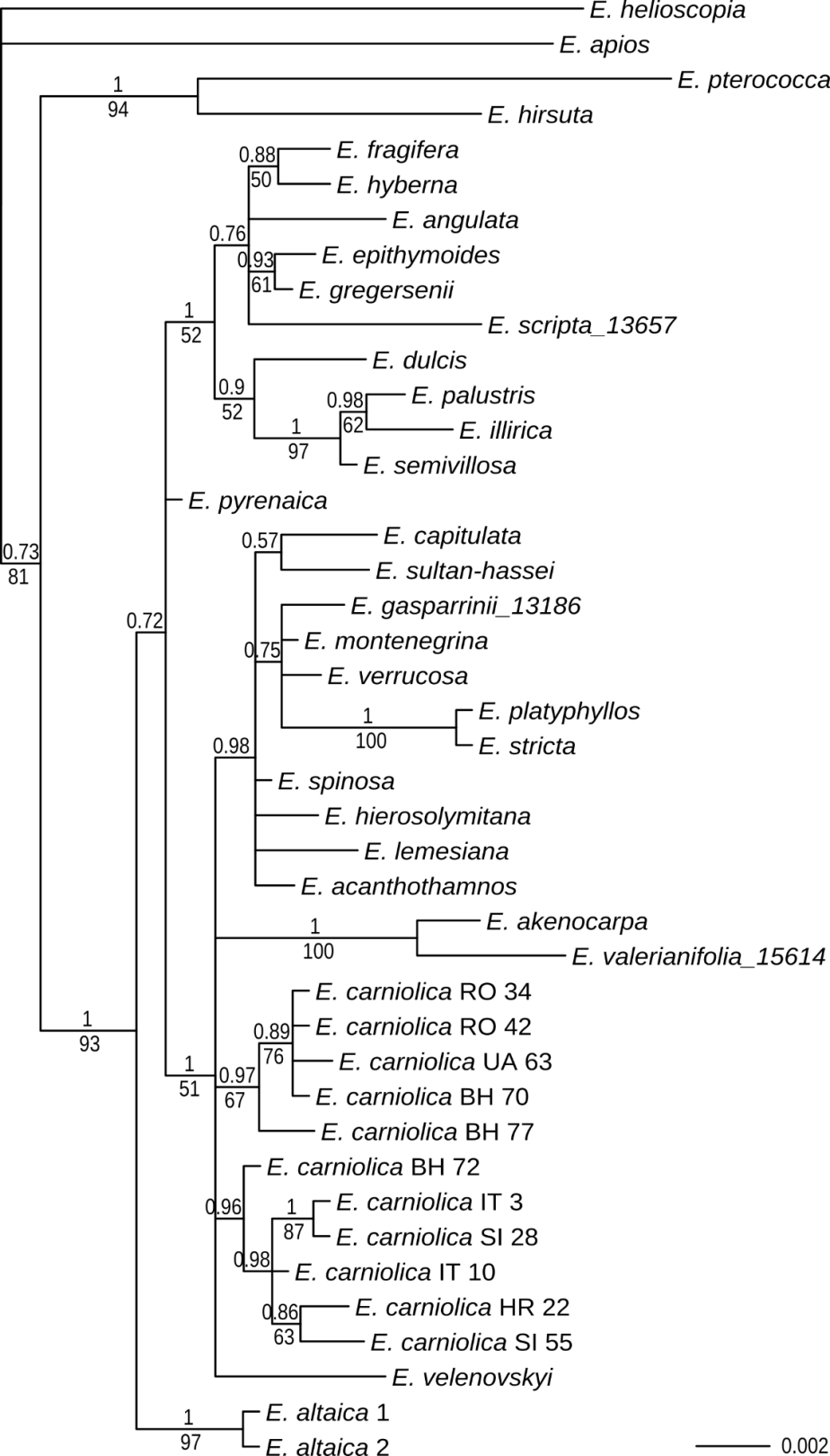


**Supplementary Figure 5.** Bayesian consensus phylogram inferred from the plastid *trnT–trnF* sequences showing phylogenetic position of *Euphorbia carniolica* within *E.* sect. *Helioscopia*. Numbers above branches are posterior probabilities > 0.5, those below branches maximum parsimony bootstrap values > 50%. Population numbers correspond to Supplementary Data 1.


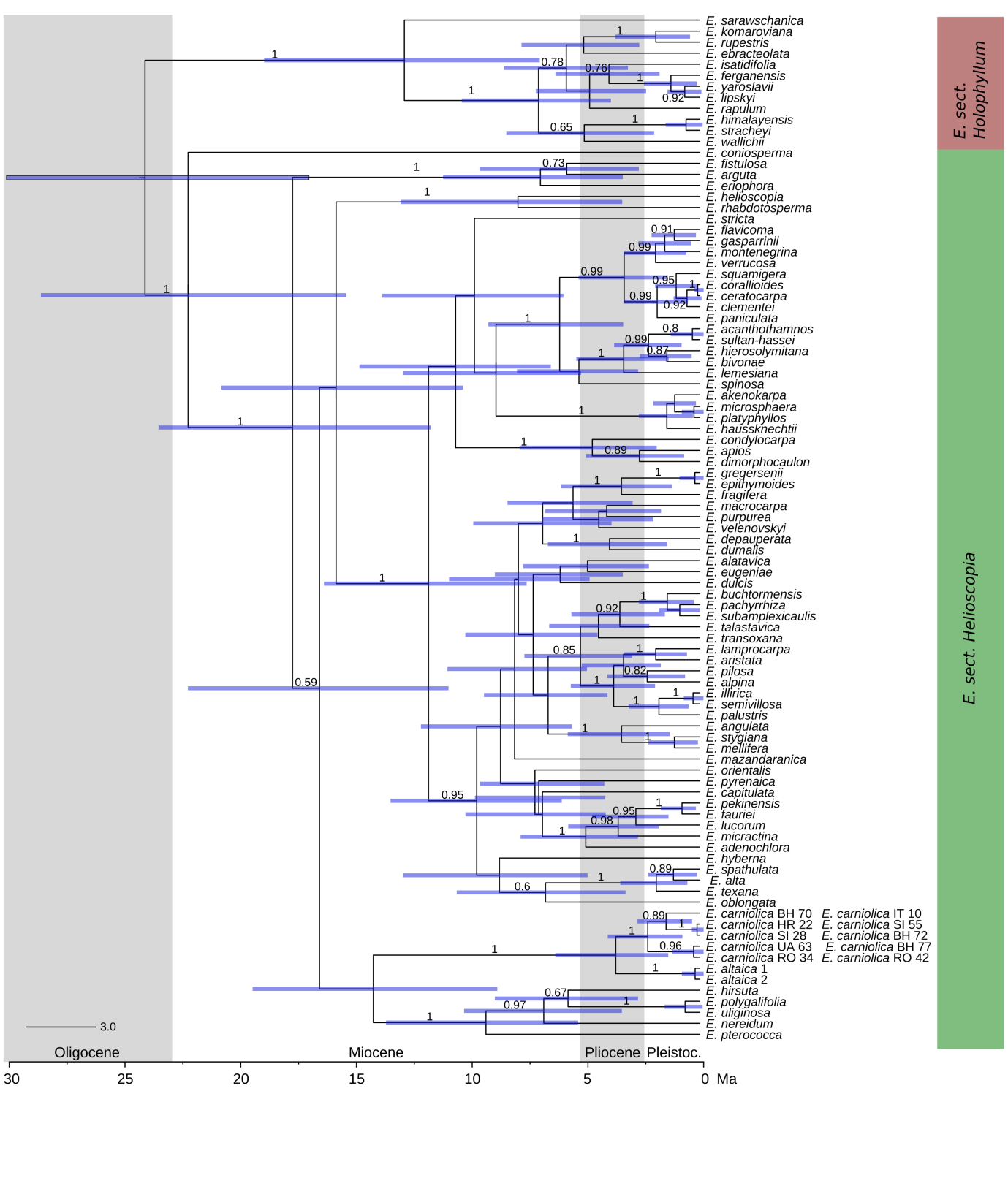
**Supplementary Figure 6.** Bayesian consensus chronogram (Maximum Clade Credibility tree) showing temporal diversification within *Euphorbia* sect. *Helioscopia*. Numbers above branches are posterior probabilities; the bars represent 95% highest posterior densities (HPD) of the age estimates. Population numbers correspond to Supplementary Data 1. In the case of *E. carniolica* populations that were resolved within the same clades in the phylogenetic analysis of the complete dataset (shown in Supplementary Figure 4) are listed in the second row.


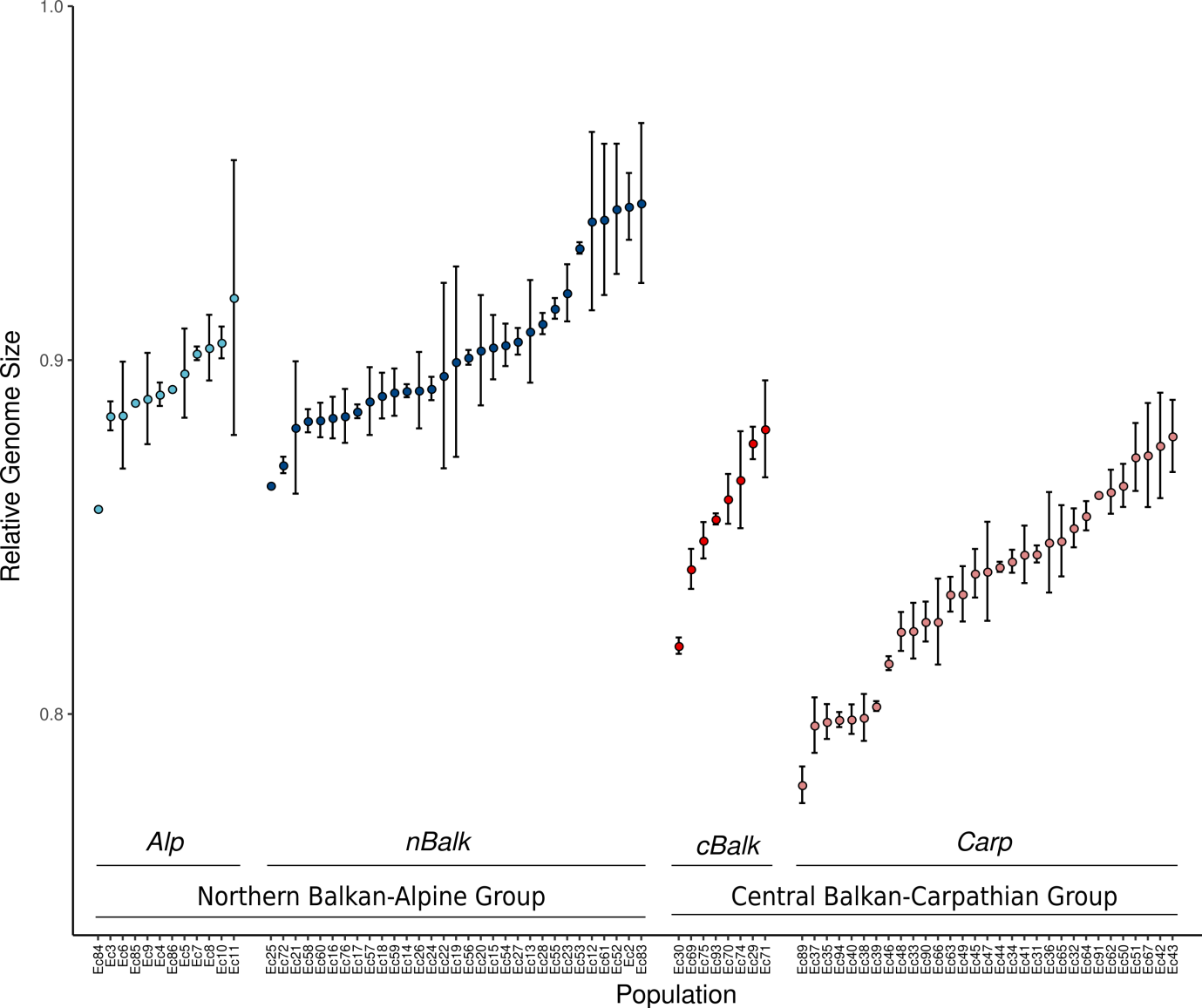
**Supplementary Figure 7.** Relative genome size (RGS) variation within different phylogroups of *Euphorbia carniolica*. Population numbers and RGS values are given in Supplementary Data 1. Shown are population mean values (dot) and standard deviation (whiskers).


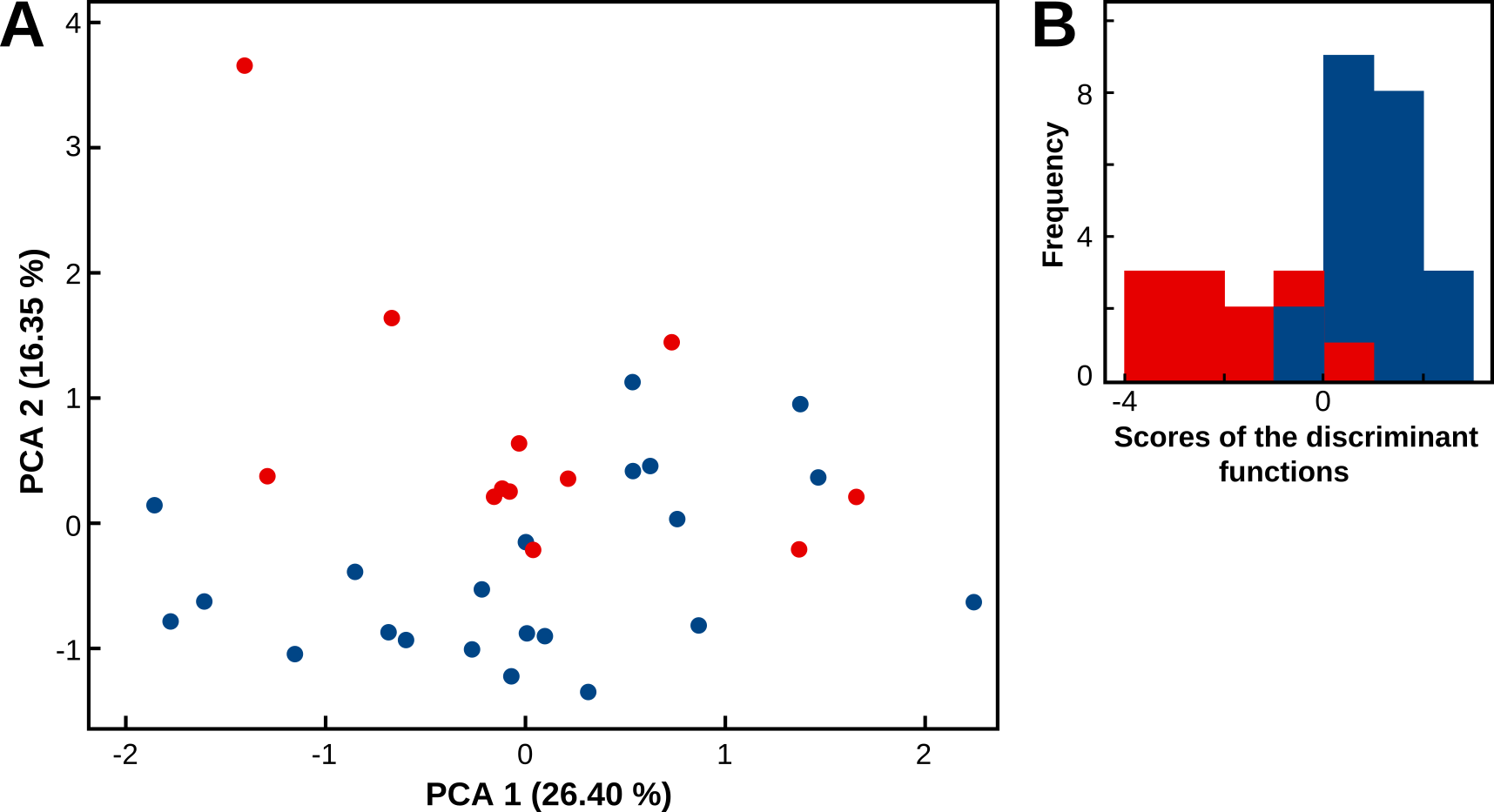


**Supplementary Figure 8.** Morphological differentiation within *Euphorbia carniolica* based on morphometric characters of cyathium and fruit characters. **A**, Principal component analysis scatter plot; **B**, histogram of discriminant analysis. Blue, *Northern Balkan-Alpine* *Group*; red, *Central Balkan-Carpthian Group*.


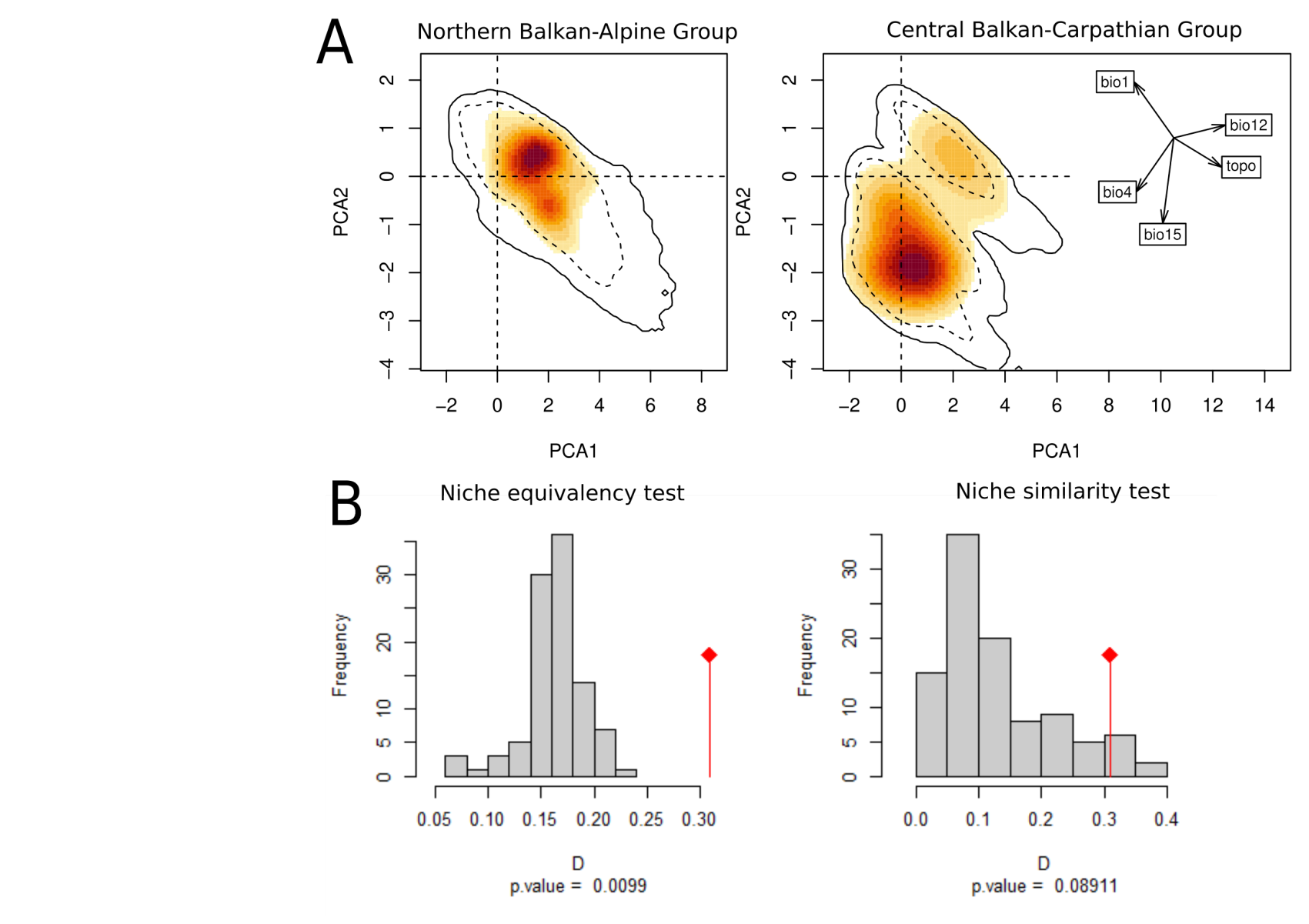


**Supplementary Figure 9**. **A,** Niche of the *Northern Balkan-Alpine Group* (left) and the *Central Balkan-Carpathian Group* (right) of *Euphorbia carniolica* in environmental space (i.e. based on the first and second axis of a PCA using four bioclimatic variables and a topographic variable) corrected by the prevalence of the environments. Shading shows the density of the occurrences of the species. The solid and dashed contour lines show 100% and 50% of the available background environment, respectively. A proportion of 44.9% and 23.2% of inertia are explained by the first and second axis of the PCA, respectively. The insert illustrates the contribution of the environmental variables: bio1, annual mean temperature; bio4, temperature seasonality; bio12, annual precipitation; bio15, precipitation seasonality; topo, standard deviation of elevations. **B**, Empirical niche overlap measured as Schöner´s D (red line) compared to the null distribution of simulated niche overlaps based on 100 replications (grey histogram). In the equivalency test (left) but not in the similarity test (right) the empirical overlap was significantly larger than the simulated one (p=0.001 and p=0.089, respectively).


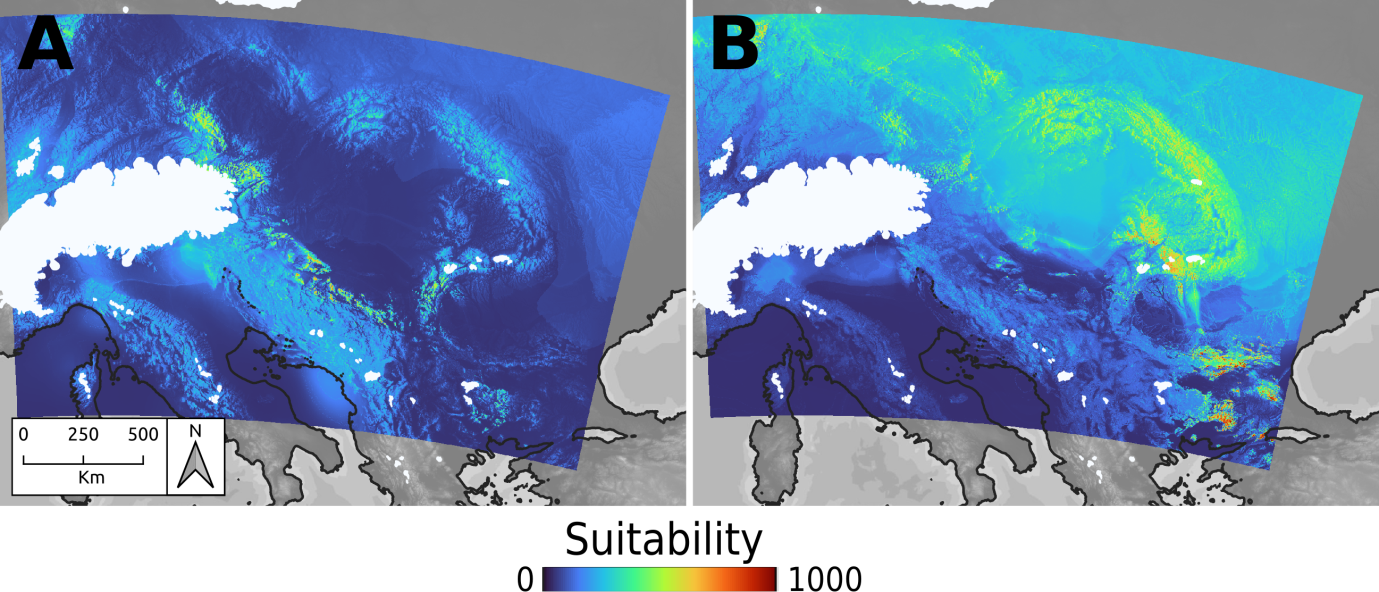
**Supplementary Figure 10.** Continuous habitat suitabilities for last glacial maximum (LGM) conditions inferred from species distribution modeling. White areas represent LGM ice cover (Ehlers et al., 2011). **A**, *Northern Balkan-Alpine Group*; **B**, *Central Balkan-Carpathian Group*.

**Supplementary Tables**

**Supplementary Table 1.** PCR primers, PCR conditions and PCR reaction mix as well as primers used for sequencing of the three different regions of *E. carniolica* and related species.

| **Region** | **PCR primers (reference)** | **PCR Primer sequence** | **PCR conditions** | **PCR Mix** | **Sequencing primers (reference)** | **Sequencing primer sequence** |
| --- | --- | --- | --- | --- | --- | --- |
| **ITS** | 17SE  (Sun et al., 1994) | ACGAATTCATGGTCCGGTGAAGTGTTCG | 5 min 94° , (30 s 94° , 30 s 56° , 1 min 72° ) x 35, decrease of 0.4° per cycle and constant temp of 48° from cycle 15, 10 min 72 | 8 μl ReadyMix (Sigma–Aldrich), 10 μl water, 0.9 μl BSA (10 mg/ml; Promega), 0.55 μl of each primer (10 μM), 1 μl of total genomic DNA | 17SE  (Sun et al., 1994) | ACGAATTCATGGTCCGGTGAAGTGTTCG |
|  | 26SE  (Sun et al., 1994) | TAGAATTCCCCGGTTCGCTCGCCGTTAC |  |  | 26SE  (Sun et al., 1994) | TAGAATTCCCCGGTTCGCTCGCCGTTAC |
| ***trnT–trnF*** | TabA  (Taberlet et al., 1991) | CATTACAAATGCGATGCTCT | 5 min 95° C, (30 s 94° C, 30 s 48° C, 4 min 65° C) x 35, 10 min 65° C | 9 μl ReadyMix (Sigma–Aldrich), 13 μl water, 1 μl BSA (10 mg/ml; Promega), 0.5 μl of each primer (10 μM), 0.5 μl of MgCl2 (25 μM, Sigma-Aldrich), 0.5–1 μl of total genomic DNA | TabA  (Taberlet et al., 1991) | CATTACAAATGCGATGCTCT |
|  |  |  |  |  | TabB  (Taberlet et al., 1991) | TCTACCGATTTCGCCATATC |
|  | TabF  (Taberlet et al., 1991) | ATTTGAACTGGTGACACGAG |  |  | TabC  (Taberlet et al., 1991) | CGAAATCGGTAGACGCTACG |
|  |  |  |  |  | TabD  (Taberlet et al., 1991) | GGGGATAGAGGGACTTGAAC |
|  |  |  |  |  | TabF  (Taberlet et al., 1991) | ATTTGAACTGGTGACACGAG |
| ***ndhF–trnL*** | ndhF  (Shaw et al., 2007)  or  ndhF_Euph3 (Pahlevani & Frajman, 2023) | GAAAGGTATKATCCAYGMATATT / GTTGTCGCCGGAAAAAGTAGAAGT | 5 min 85 °C, (30 s 94° C, 30 s 56° C, 4 min 72° C) x 35, 10 min 72° C | 1.6 µl 10x Buffer, 1.2 µl dNTPMix, 0.2 µl TaqDNA polymerase (all TaKaRa), 0.6 µl of both primers (10 µM), 1µl DNA template | ndhF_Euph2  (Pahlevani &  Frajman, 2023) | CCATGAATATTGATATATATGTTCC |
|  | trnL(UAG)  (Shaw et al., 2007) | CTGCTTCCTAAGAGCAGCGT |  |  |  |  |

**Supplementary Table 2.** Outgroup GenBank accession numbers of ITS and *trnT–trnF* sequences.

|  | **Taxon** | **ITS** | ***trnT–trnF*** |
| --- | --- | --- | --- |
| 1 | *Euphorbia acanthothamnos* Heldr. ex Sart. | KT071806 | KT071795 |
| 2 | *Euphorbia adenochlora* C. Morren & Decne. | KC212161 |  |
| 3 | *Euphorbia akenocarpa* Guss. | JN010023 | JN009923 |
| 4 | *Euphorbia alatavica* Boiss. | GU953741 |  |
| 5 | *Euphorbia alpina* Ledeb. | KC212168 |  |
| 6 | *Euphorbia alta* Norton | AF537553 |  |
| 7 | *Euphorbia altaica* C.A.Mey. ex Ledeb. 1 | OQ519864 | 16635 |
| 8 | *Euphorbia altaica* C.A.Mey. ex Ledeb. 2 | OQ519865 | 16636 |
| 9 | *Euphorbia altaica* C.A.Mey. ex Ledeb. 3 | KC212169 |  |
| 10 | *Euphorbia altaica* C.A.Mey. ex Ledeb. 4 | GU979429 |  |
| 11 | *Euphorbia altaica* C.A.Mey. ex Ledeb. 5 | KC212170 |  |
| 12 | *Euphorbia angulata* Jacq. | JN010026 | JN009925 |
| 13 | *Euphorbia apios* L. | JN010027 | JN009927 |
| 14 | *Euphorbia arguta* Banks & Sol. | KC212176 |  |
| 15 | *Euphorbia aristata* Schmalh. | GU979434 |  |
| 16 | *Euphorbia bivonae* Steud. | KT071809 |  |
| 17 | *Euphorbia buchtormensis* Ledeb. | KC212196 |  |
| 18 | *Euphorbia capitulata* Rchb. | JN010032 | JN009932 |
| 19 | *Euphorbia ceratocarpa* Ten. | OQ519875 |  |
| 20 | *Euphorbia clementei* Boiss. | KC212206 |  |
| 21 | *Euphorbia condylocarpa* M. Bieb. | KC212208 |  |
| 22 | *Euphorbia coniosperma* Boiss. & Buhse | KC212209 |  |
| 23 | *Euphorbia corallioides* L. | HQ900597 |  |
| 24 | *Euphorbia depauperata* Hochst. ex A. Rich. | KC212226 |  |
| 25 | *Euphorbia dimorphocaulon* P.H. Davis | OQ519876 |  |
| 26 | *Euphorbia dulcis* L. | JN010041 | JN009942 |
| 27 | *Euphorbia dumalis* S. Carter | KC212232 |  |
| 28 | *Euphorbia ebracteolata* Hayata | EU659768 |  |
| 29 | *Euphorbia epithymoides* L. | JN010082 | JN009984 |
| 30 | *Euphorbia eriophora* Boiss. | KC212238 |  |
| 31 | *Euphorbia eugeniae* Prokh. | GU979428 |  |
| 32 | *Euphorbia fauriei* H. Lév. & Vaniot | EU659765 |  |
| 33 | *Euphorbia ferganensis* B. Fedtsch. | KC212248 |  |
| 34 | *Euphorbia fistulosa* M.S. Khan | KC212249 |  |
| 35 | *Euphorbia flavicoma* DC. | MT957509 |  |
| 36 | *Euphorbia fragifera* Jan | JN010048 | JN009949 |
| 37 | *Euphorbia gasparrinii* Boiss. | MK088031 | 13186 |
| 38 | *Euphorbia gregersenii* K. Maly ex Beck | JN010051 | JN009952 |
| 39 | *Euphorbia haussknechtii* Boiss. | KC212269 |  |
| 40 | *Euphorbia helioscopia* L. | JN010052 | JN009953 |
| 41 | *Euphorbia hierosolymitana* Boiss. | KT071813 | KT071798 |
| 42 | *Euphorbia himalayensis* (Klotzsch) Boiss. | KC212392 |  |
| 43 | *Euphorbia hirsuta* L. | JN010055 | JN009956 |
| 44 | *Euphorbia hyberna* L. | JN010056 | JN009957 |
| 45 | *Euphorbia illirica* Lam. | JN010116 | JN010017 |
| 46 | *Euphorbia isatidifolia* Lam. | HQ900617 |  |
| 47 | *Euphorbia komaroviana* Prokh. | EU659770 |  |
| 48 | *Euphorbia lamprocarpa* Prokh. | OQ519877 |  |
| 49 | *Euphorbia lemesiana* Hadjik., Hand., Christodoulou & Frajman | KT071814 | KT071801 |
| 50 | *Euphorbia lipskyi* (Prokh.) Prokh. | KC212295 |  |
| 51 | *Euphorbia lucorum* Rupr. | EU659771 |  |
| 52 | *Euphorbia macrocarpa* Boiss. & Buhse | KC212297 |  |
| 53 | *Euphorbia mazandaranica* Pahlevani | KC212304 |  |
| 54 | *Euphorbia mellifera* Aiton | KC212306 |  |
| 55 | *Euphorbia micractina* Boiss. | KC212308 |  |
| 56 | *Euphorbia microsphaera* Boiss. | KC212313 |  |
| 57 | *Euphorbia montenegrina* (Bald.) K. Maly ex Rohlena | JN010068 | JN009969 |
| 58 | *Euphorbia nereidum* Jahand. & Maire | JN250198 |  |
| 59 | *Euphorbia oblongata* Griseb. | OQ519878 |  |
| 60 | *Euphorbia orientalis* L. | EU659764 |  |
| 61 | *Euphorbia pachyrrhiza* Kar. & Kir. | KC212328 |  |
| 62 | *Euphorbia palustris* L. | JN010073 | JN009974 |
| 63 | *Euphorbia paniculata* Desf. | HQ900639 |  |
| 64 | *Euphorbia pekinensis* Rupr. | EU659766 |  |
| 65 | *Euphorbia pilosa* L. | KC212337 |  |
| 66 | *Euphorbia platyphyllos* L. | JN010109 | JN010010 |
| 67 | *Euphorbia polygalifolia* Boiss. & Reut. | KC212343 |  |
| 68 | *Euphorbia pterococca* Brot. | JN010085 | JN009986 |
| 69 | *Euphorbia purpurea* (Raf.) Fernald | KC212349 |  |
| 70 | *Euphorbia pyrenaica* Jord. | JN010035 | JN009935 |
| 71 | *Euphorbia rapulum* Kar. & Kir. | KC212357 |  |
| 72 | *Euphorbia rhabdotosperma* Radcl.-Sm. | KC212361 |  |
| 73 | *Euphorbia rupestris* Ledeb. | KC212364 |  |
| 74 | *Euphorbia sarawschanica* Regel | GU979438 |  |
| 75 | *Euphorbia scripta* Somm. & Lev. |  | 13657 |
| 76 | *Euphorbia semivillosa* Prokh. | JN010098 | JN009999 |
| 77 | *Euphorbia spathulata* Lam. | JN250242 |  |
| 78 | *Euphorbia spinosa* L. | JN010101 | JN010003 |
| 79 | *Euphorbia squamigera* Loisel. | HQ900657 |  |
| 80 | *Euphorbia stracheyi* Boiss. | KC212389 |  |
| 81 | *Euphorbia stricta* L. | JN010104 | JN010005 |
| 82 | *Euphorbia stygiana* H.C. Watson | KC212397 |  |
| 83 | *Euphorbia subamplexicaulis* Kar. & Kir. | KC212398 |  |
| 84 | *Euphorbia sultan-hassei* Strid., Bentzer et al. | KT071818 | KT071805 |
| 85 | *Euphorbia talastavica* (Prokh.) Prokh. | KC212405 |  |
| 86 | *Euphorbia texana* Boiss. | KC212409 |  |
| 87 | *Euphorbia transoxana* (Prokh.) Prokh. | GU979425 |  |
| 88 | *Euphorbia uliginosa* Welw. ex Boiss. | KC212418 |  |
| 89 | *Euphorbia valerianifolia* Lam. | JN010109 | 15614 |
| 90 | *Euphorbia velenovskyi* Bornm. | JN010113 | JN010015 |
| 91 | *Euphorbia verrucosa* L. | JN010115 | JN010016 |
| 92 | *Euphorbia wallichii* Hook.f. | KC212426 |  |
| 93 | *Euphorbia yaroslavii* Poljakov | KC212429 |  |

**Supplementary Table 3.** Names, descriptions and references for each model included in the demographic model selection.

| **Model name** | **Model description** | **Reference for model** |
| --- | --- | --- |
| founder_asym | Split into two populations, with two migration rates | Charles et al. (2018) |
| founder_anc_asym_two_epoch | Split into two populations in first epoch via founder event with exponential growth and continuous asymmetric migration followed a second epoch with no exponential growth and no migration | Zaveska et al. 2021 |
| founder_anc_sym_two_epoch | Split into two populations in first epoch via founder event with exponential growth and continuous symmetric migration followed a second epoch with no exponential growth and no migration | Zaveska et al. 2021 |
| founder_sec_contact_asym_two_epoch | Split into two populations in first epoch via founder event with exponential growth and no migration followed by a second epoch with no exponential growth and asymmetric migration | Zaveska et al. 2021 |
| founder_sym | Split into two populations, with one migration rate | Charles et al. (2018) |
| vic_two_epoch_admix | Split into two populations, no migration but a discrete admixture event from pop 1 into pop 2 occurs (between two drift events) | Charles et al. (2018) |
| vic_sec_contact_asym_mig | Split with no gene flow, followed by period of asymmetrical gene flow | Charles et al. (2018) |
| vic_anc_asym_mig | Split with asymmetric migration followed by isolation | Charles et al. (2018) |
| vic_no_mig_admix_late | Split into two populations, no migration but a discrete admixture event from pop 1 into pop 2 occurs (after drift) | Charles et al. (2018) |
| founder_nomig_admix_two_epoch | Split into two populations, no migration but a discrete admixture event from pop 1 into pop 2 occurs (between two drift events) | Charles et al. (2018) |
| founder_nomig_admix_first_epoch | Split into two populations in first epoch via founder event and unidirectional discrete admixture event occurring at beginning of the first epoch; no exponential growth and no migration in second epoch | Zaveska et al. 2021 |
| vic_no_mig | Split into two populations, no migration | Charles et al. (2018) |
| founder_no_mig_two_epoch | Split into two populations in first epoch via founder event with exponential growth and no migration followed a second epoch without exponential growth and no migration. | Zaveska et al. 2021 |
| vic_no_mig_admix_early | Split into two populations, no migration but a discrete admixture event from pop 1 into pop 2 occurs (before drift) | Charles et al. (2018) |
| founder_nomig | Split into two populations, with no migration | Charles et al. (2018) |
| founder_nomig_admix_late | Split into two populations, no migration but a discrete admixture event from pop 1 into pop 2 occurs (after drift) | Charles et al. (2018) |
| founder_nomig_admix_early | Split into two populations, no migration but a discrete admixture event from pop 1 into pop 2 occurs (before drift) | Charles et al. (2018) |
| founder_nomig | Split into two populations, with no migration | Charles et al. (2018) |

**Supplementary Table 4.** Characters studied in the morphometric analyses of *Euphorbia carniolica*.

| **No.** | **Total plant** |
| --- | --- |
| 1 | Plant height, cm |
|  | **Stem** |
| 2 | Stem length, cm |
| 3 | Stem width, mm |
| 4 | Ratio Stem length /Plant height |
| 5 | Number of trichomes along 1 cm × 1 mm in the middle of the lower half of the stem |
|  | **Axillary and terminal rays** |
| 6 | Number of axillary rays |
| 7 | Length of the longest axillary ray, cm |
| 8 | Length of the stem from the basis to the lowest axillary ray, cm |
| 9 | Ratio Length of the stem from the basis to the lowest axillary ray / Stem length |
| 10 | Number of terminal rays |
| 11 | Length of the terminal rays, cm |
| 12 | Number of branchings of terminal rays |
| 13 | Ratio Length of the terminal rays / Plant height |
|  | **Middle stem leaves** |
| 14 | Length of the middle stem leaf, mm |
| 15 | Width of the middle stem leaf, mm |
| 16 | Ratio Length / Width of a middle stem leaf |
| 17 | Distance from the base to the widest part of a middle stem leaf |
| 18 | Ratio of Distance from the base to the widest part of a middle stem leaf / Length of a middle stem leaf |
| 19 | Length of the leaf petiole |
| 20 | Angle of the leaf tip, (°) |
| 21 | Area of a middle stem leaf, mm² |
| 22 | Outline of a middle stem leaf |
| 23 | Number of trichomes in 1 mm² of the upper leaf surface |
| 24 | Number of trichomes in 1 mm² of the lower leaf surface |
| 25 | Length of 3 trichomes on 1 mm² upper leaf surface |
| 26 | Length of 3 trichomes on 1 mm² lower leaf surface |
|  | **Ray leaves** |
| 27 | Length of a ray leaf, mm |
| 28 | Width of a ray leaf, mm |
| 29 | Ratio Length / Width of a ray leaf |
| 30 | Distance from the base to the widest part of a ray leaf, mm |
| 31 | Ratio of Distance from the base to the widest part of a ray leaf / Length of a ray leaf |
| 32 | Angle of the tip of a ray leaf (°) |
| 33 | Area of a ray leaf square, mm² |
| 34 | Outline of a ray leaf |
|  | **Raylet leaves** |
| 35 | Length of a raylet leaf, mm |
| 36 | Width of a raylet leaf, mm |
| 37 | Ratio length/width of a raylet leaf |
| 38 | Distance from the base to the widest part of a raylet leaf, mm |
| 39 | Ratio of Distance from the base to the widest part of a raylet leaf / Length of a raylet leaf |
| 40 | Angle of tip of a raylet leaf, (°) |
| 41 | Area of a raylet leaf square, mm² |
| 42 | Outline of a raylet leaf |
|  | **Cyathium** |
| 43 | Length of cyathial involucre, mm |
| 44 | Width of cyathial involucre, mm |
| 45 | Ratio Length / Width of cyathial involucre |
| 46 | Length of the central cyathial pedicel |
| 47 | Length of a terminal cyathial pedicel |
| 48 | Length of cyathial gland, mm |
| 49 | Width of cyathial gland, mm |
| 50 | Ratio Length / Width of cyathial gland |
|  | **Fruits** |
| 51 | Fruit length, mm |
| 52 | Fruit width, mm |
| 53 | Ratio Fruit length / Fruit width |
| 54 | Distance from the base to the widest part of the fruit, mm |
| 55 | Ratio of Distance from the base to the widest part of the fruit / Fruit length |
| 56 | Length of a wart on the fruit, mm |
| 57 | Width of a wart on the fruit, mm |
| 58 | Ratio Length / width of a wart on the fruit |
| 59 | Distance from the base to the widest part of a wart, mm |
| 60 | Ratio of Distance from the base to the widest part of a wart / Length of a wart on the fruit |
| 61 | Style length, mm |
|  | **Seeds** |
| 62 | Seed length, mm |
| 63 | Seed width, mm |
| 64 | Ratio Seed length / Seed width |
| 65 | Distance from the base to the widest part of a seed, mm |
| 66 | Ratio Distance from the base to the widest part of a seed / Seed length |
| 67 | Caruncle length, mm |
| 68 | Caruncle width mm |
| 69 | Ratio Caruncle length / Caruncle width |
| 70 | Distance from the base to the widest part of caruncle, mm |
| 71 | Ratio of Distance from the base to the widest part of caruncle / Caruncle length |

| **Supplementary Table 5.** Results from demographic modeling for the genetic groups *nBalk* vs. *cBalk*, *nBalk* vs. *Alps* and *cBalk* vs *Carp*. Models are ordered starting with the best fit. The employed models are from Charles et al. (2018) and Záveská et al. (2021), and abbreviations and model definitions correspond to these publications. AIC, Akaike information criterion; ΔAIC, difference of AIC to the best-scoring model; ω_i_, Akaike weight calculated incorporating all models; theta, effective mutation rate of the reference population. | | | | | | | |
| --- | --- | --- | --- | --- | --- | --- | --- |
| ***nBalk* versus *cBalk*** | | | | | | | |
| Directionality (Mainland vs. Island) | Model | Log-likelihood | AIC | ΔAIC | ω_i_ | Chi-squared | Theta |
| nBalk vs. cBalk | vic_sec_contact_asym_mig | -479.14 | 974.28 | 0 | 1.00 | 381.97 | 260.6 |
| cBalk vs. nBalk | vic_sec_contact_asym_mig | -488.03 | 992.06 | 17.78 | 0.00 | 441.17 | 228.3 |
| nBalk vs. cBalk | vic_two_epoch_admix | -504.18 | 1022.36 | 48.08 | 0.00 | 693.97 | 297.86 |
| cBalk vs. nBalk | vic_two_epoch_admix | -510.6 | 1035.2 | 60.92 | 0.00 | 702.8 | 239.87 |
| nBalk vs. cBalk | vic_anc_asym_mig | -509.67 | 1035.34 | 61.06 | 0.00 | 611.94 | 283.83 |
| nBalk vs. cBalk | vic_no_mig_admix_late | -512.28 | 1036.56 | 62.28 | 0.00 | 588.15 | 296.84 |
| cBalk vs. nBalk | vic_no_mig_admix_late | -514.94 | 1041.88 | 67.6 | 0.00 | 579.37 | 291.83 |
| cBalk vs. nBalk | vic_anc_asym_mig | -530.05 | 1076.1 | 101.82 | 0.00 | 1018.21 | 281.99 |
| cBalk vs. nBalk | founder_anc_asym_two_epoch | -567.63 | 1151.26 | 176.98 | 0.00 | 1831.98 | 203.89 |
| nBalk vs. cBalk | founder_anc_asym_two_epoch | -568.73 | 1153.46 | 179.18 | 0.00 | 1531.46 | 209.6 |
| nBalk vs. cBalk | founder_asym | -587.89 | 1189.78 | 215.5 | 0.00 | 987.89 | 1690.49 |
| cBalk vs. nBalk | founder_asym | -590.22 | 1194.44 | 220.16 | 0.00 | 822.99 | 74.55 |
| cBalk vs. nBalk | vic_no_mig | -600.99 | 1211.98 | 237.7 | 0.00 | 1127.51 | 344.27 |
| nBalk vs. cBalk | vic_no_mig | -601 | 1212 | 237.72 | 0.00 | 1182.32 | 343.91 |
| cBalk vs. nBalk | vic_no_mig_admix_early | -601.02 | 1214.04 | 239.76 | 0.00 | 1116.46 | 344.23 |
| nBalk vs. cBalk | vic_no_mig_admix_early | -601.33 | 1214.66 | 240.38 | 0.00 | 1176.52 | 337.42 |
| nBalk vs. cBalk | founder_anc_sym_two_epoch | -612.81 | 1239.62 | 265.34 | 0.00 | 1217.28 | 326.65 |
| nBalk vs. cBalk | founder_sec_contact_asym_two_epoch | -659.06 | 1334.12 | 359.84 | 0.00 | 2200.6 | 129.52 |
| cBalk vs. nBalk | founder_sec_contact_asym_two_epoch | -662.69 | 1341.38 | 367.1 | 0.00 | 1868.83 | 919.61 |
| nBalk vs. cBalk | founder_sym | -697.13 | 1406.26 | 431.98 | 0.00 | 860.22 | 60.06 |
| cBalk vs. nBalk | founder_sym | -697.96 | 1407.92 | 433.64 | 0.00 | 844.42 | 164 |
| cBalk vs. nBalk | founder_anc_sym_two_epoch | -723.21 | 1460.42 | 486.14 | 0.00 | 1428.25 | 202.39 |
| cBalk vs. nBalk | founder_nomig_admix_two_epoch | -876.05 | 1766.1 | 791.82 | 0.00 | 1097.14 | 97.2 |
| nBalk vs. cBalk | founder_nomig_admix_two_epoch | -877.35 | 1768.7 | 794.42 | 0.00 | 1155.97 | 119.69 |
| cBalk vs. nBalk | founder_no_mig_two_epoch | -900.56 | 1813.12 | 838.84 | 0.00 | 1193.65 | 69.54 |
| nBalk vs. cBalk | founder_no_mig_two_epoch | -901.43 | 1814.86 | 840.58 | 0.00 | 1169.03 | 82.18 |
| nBalk vs. cBalk | founder_nomig_admix_first_epoch | -906.34 | 1826.68 | 852.4 | 0.00 | 1190.35 | 235.96 |
| cBalk vs. nBalk | founder_nomig_admix_first_epoch | -908.88 | 1831.76 | 857.48 | 0.00 | 1194.56 | 183.06 |
| cBalk vs. nBalk | founder_nomig_admix_late | -911.16 | 1834.32 | 860.04 | 0.00 | 1103.54 | 900.82 |
| nBalk vs. cBalk | founder_nomig_admix_late | -913.87 | 1839.74 | 865.46 | 0.00 | 1105.91 | 660.2 |
| cBalk vs. nBalk | founder_nomig | -918.29 | 1846.58 | 872.3 | 0.00 | 1207.54 | 4979.03 |
| nBalk vs. cBalk | founder_nomig | -927.21 | 1864.42 | 890.14 | 0.00 | 1209.15 | 2085.32 |
| nBalk vs. cBalk | founder_nomig_admix_early | -933.37 | 1878.74 | 904.46 | 0.00 | 1222.21 | 2230.81 |
| cBalk vs. nBalk | founder_nomig_admix_early | -939.87 | 1891.74 | 917.46 | 0.00 | 1223.11 | 1172.68 |

| ***nBalk* versus *Alps*** | |  |  |  |  |  |
| --- | --- | --- | --- | --- | --- | --- |
| Model | Log-likelihood | AIC | ΔAIC | ω_i_ | Chi-squared | Theta |
| founder_sym | -871.05 | 1754.1 | 0 | 0.99 | 589.3 | 950.9 |
| founder_asym | -874.61 | 1763.22 | 9.12 | 0.01 | 585.05 | 1322.49 |
| founder_anc_sym_two_epoch | -885.5 | 1785 | 30.9 | 0.00 | 610.92 | 1014.82 |
| founder_sec_contact_asym_two_epoch | -886.02 | 1788.04 | 33.94 | 0.00 | 584.2 | 1071.83 |
| vic_two_epoch_admix | -985.38 | 1984.76 | 230.66 | 0.00 | 817.45 | 1311.14 |
| vic_no_mig_admix_late | -1016.4 | 2044.8 | 290.7 | 0.00 | 972.04 | 1573.18 |
| vic_anc_asym_mig | -1043.64 | 2103.28 | 349.18 | 0.00 | 1054.87 | 1533.05 |
| vic_sec_contact_asym_mig | -1049.62 | 2115.24 | 361.14 | 0.00 | 854.38 | 1394.71 |
| founder_anc_asym_two_epoch | -1165.16 | 2346.32 | 592.22 | 0.00 | 1215.76 | 1808.15 |
| founder_nomig_admix_two_epoch | -1182.59 | 2379.18 | 625.08 | 0.00 | 1417.18 | 5042.26 |
| founder_no_mig_two_epoch | -1292.86 | 2597.72 | 843.62 | 0.00 | 1237.39 | 776.25 |
| founder_nomig_admix_first_epoch | -1337.79 | 2689.58 | 935.48 | 0.00 | 1298.92 | 1658.38 |
| founder_nomig_admix_late | -1357.68 | 2727.36 | 973.26 | 0.00 | 1272.58 | 28874.95 |
| vic_no_mig | -1435.1 | 2880.2 | 1126.1 | 0.00 | 2165.69 | 1870.43 |
| vic_no_mig_admix_early | -1435.74 | 2883.48 | 1129.38 | 0.00 | 2218.62 | 1888.54 |
|  | | | | | | |
| ***cBalk* versus *Carp*** |  |  |  |  |  |  |
| Model | Log-likelihood | AIC | ΔAIC | ω_i_ | Chi-squared | Theta |
| founder_asym | -431.95 | 877.9 | 0 | 1.00 | 136.4 | 1788.7 |
| founder_anc_asym_two_epoch | -439.61 | 895.22 | 17.32 | 0.00 | 148.62 | 544.32 |
| founder_anc_sym_two_epoch | -487.27 | 988.54 | 110.64 | 0.00 | 254.55 | 798.36 |
| founder_sec_contact_asym_two_epoch | -489.09 | 994.18 | 116.28 | 0.00 | 240.28 | 425.87 |
| founder_sym | -524.52 | 1061.04 | 183.14 | 0.00 | 312.48 | 3884.21 |
| vic_two_epoch_admix | -575.24 | 1164.48 | 286.58 | 0.00 | 534.4 | 364.97 |
| vic_sec_contact_asym_mig | -595.8 | 1207.6 | 329.7 | 0.00 | 648.3 | 343.24 |
| vic_anc_asym_mig | -626.46 | 1268.92 | 391.02 | 0.00 | 655.72 | 298.08 |
| vic_no_mig_admix_late | -645.38 | 1302.76 | 424.86 | 0.00 | 842.26 | 407.67 |
| founder_nomig_admix_two_epoch | -720.64 | 1455.28 | 577.38 | 0.00 | 782.58 | 1506.4 |
| founder_nomig_admix_first_epoch | -839.83 | 1693.66 | 815.76 | 0.00 | 978.56 | 1172.39 |
| vic_no_mig | -853.4 | 1716.8 | 838.9 | 0.00 | 1116.54 | 541.8 |
| founder_no_mig_two_epoch | -853.06 | 1718.12 | 840.22 | 0.00 | 1035.76 | 372.07 |
| vic_no_mig_admix_early | -853.91 | 1719.82 | 841.92 | 0.00 | 1107.95 | 554.13 |
| founder_nomig | -986.01 | 1982.02 | 1104.12 | 0.00 | 1225.01 | 10334.15 |
| founder_nomig_admix_late | -998.69 | 2009.38 | 1131.48 | 0.00 | 1156.13 | 2348.24 |
| founder_nomig_admix_early | -1009.61 | 2031.22 | 1153.32 | 0.00 | 1276.41 | 10688.85 |
| founder_nomig | -1551.92 | 3113.84 | 1359.74 | 0.00 | 1657.34 | 12335.95 |

**REFERENCE**

Ehlers, J., Gibbard, P. L., & Hughes, P. D. (2011). Quaternary glaciations – Extent and chronology: A closer look (Vol. 15). Elsevier.

Pahlevani, A. H., & Frajman, B. (2023). Widespread, but less than assumed: Populations of Euphorbia amygdaloides (Euphorbiaceae) from western Asia represent two new cryptic species. Perspectives in Plant Ecology, Evolution and Systematics, 58, 125717.

Shaw, J., Lickey, E. B., Schilling, E. E., & Small, R. L. (2007). Comparison of whole chloroplast genome sequences to choose noncoding regions for phylogenetic studies in angiosperms: The tortoise and the hare III. American Journal of Botany, 94(3), 275–288. https://doi.org/10.3732/ajb.94.3.275

Sun, Y., Skinner, D. Z., Liang, G. H., & Hulbert, S. H. (1994). Phylogenetic analysis of Sorghum and related taxa using internal transcribed spacers of nuclear ribosomal DNA. Theoretical and Applied Genetics, 89(1), 26–32. https://doi.org/10.1007/BF00226978

Taberlet, P., Gielly, L., Pautou, G., & Bouvet, J. (1991). Universal primers for amplification of three non-coding regions of chloroplast DNA. Plant Molecular Biology, 17(5), 1105–1109. https://doi.org/10.1007/BF00037152
